# Modelling collective action to change social norms around domestic violence: social dilemmas and the role of altruism

**DOI:** 10.1057/s41599-021-00730-z

**Published:** 2021-03-01

**Authors:** Lu Gram, Rolando Granados, Eva M. Krockow, Nayreen Daruwalla, David Osrin

**Affiliations:** 1 Institute for Global Health, University College London, London, UK; 2 Department of Neuroscience, Psychology and Behaviour, University of Leicester, Leicester, UK; 3 Prevention of Violence against Women and Children, Society for Nutrition, Education and Health Action (SNEHA), Mumbai, India

## Abstract

Interventions promoting collective action have been used to prevent domestic violence in a range of settings, but their mechanisms of operation remain unclear. We formalise and combine feminist theoretical approaches to domestic violence into a game-theoretic model of women’s collective action to change gendered social norms and outcomes. We show that social norms create a social dilemma in which it is individually rational for women to abstain from action to prevent domestic violence among neighbours, but all women suffer negative consequences if none take action. Promoting altruism among women can overcome the social dilemma. Discouraging women from tolerating domestic violence, imposing additional external punishment on men for perpetrating violence, or lowering costs to women of taking action against violence may not work or even backfire. We invite researchers on community mobilisation to use our framework to frame their understandings of collective action to prevent domestic violence.

## Introduction

Worldwide, domestic violence is a critical concern for virtually all aspects of society, with severe human, emotional, and economic costs (Garcia-Moreno and Watts, 2011). One form of domestic violence, intimate partner violence, is estimated to affect 30% of women at least once in their lifetime, and is an important cause of mental, physical, and reproductive harm (Devries et al., 2013; Dillon et al., 2013; Hill et al., 2016). International declarations including the Sustainable development goals have committed national governments to eliminating domestic violence (UN, 2017). However, investments in prevention and services for survivors of violence remain inadequate (Garcia-Moreno and Watts, 2011).

Community mobilisation interventions have long been of interest to policymakers and practitioners as a means of addressing otherwise intractable societal and environmental barriers to improving health (Rosato et al., 2008). They can be defined as interventions in which local individuals collaborate with external agents in identifying, prioritising, and tackling the causes of ill-health based on principles of bottom-up leadership and empowerment (Rosato et al., 2008). For example, interventions in South Africa and Uganda have trained volunteer activists to take action against domestic violence, engaged community groups in reflection and action on gender norms, and organised large-scale campaigns and marches (Abramsky et al., 2014; Pronyk et al., 2006; Wagman et al., 2015).

Randomised controlled trials have shown that such interventions can reduce domestic violence in contexts of severe poverty and gender inequality (Bourey et al., 2015). However, the mechanism through which interventions achieve impact remains poorly understood (Gram et al., 2019). Existing theoretical frameworks display ‘positive a priori bias’ (Abimbola, 2019), in which interventions are assumed to produce positive engagement with communities without complications. The failure of recent interventions in Rwanda (Chatterji et al., 2020), Afghanistan (Gibbs et al., 2018), and Nepal (Clark et al., 2020) to show comparable impacts on domestic violence to those observed in Uganda (Abramsky et al., 2014; Wagman et al., 2015) underlines the need to understand contexts and mechanisms.

Community mobilisation interventions are complex interventions that involve long causal chains from implementation to outcome, multiple recursive feedback loops, and emergent outcomes (Anderson, 2008). Social scientists often use mathematical models to make explicit their assumptions behind implicit, verbal models of social phenomena (Epstein, 2008; Heckman, 2005; Oliver and Myers, 2002). This enables them to derive predictions using algebra or computer simulation, check for logical consistency of verbal explanations, and illuminate core uncertainties in existing evidence. Here, the goal is not to forecast the future or mirror reality as closely as possible, but rather to generate explanatory clarity (Epstein, 2008). Intervention researchers have proposed mathematical models as a tool to study the system dynamics of complex public health interventions (Davey et al., 2018).

We use a game-theoretic model to formalise oft-hypothesised processes of action to challenge unequal gender norms in community mobilisation interventions in low- and middle-income contexts. In so doing, we follow an established tradition in the social sciences of using mathematical models as thought experiments to generate logically consistent explanations for social phenomena (Centola et al., 2005; Oliver, 1993). We show how we can define conditions under which the prevention of domestic violence takes on the characteristics of a *social dilemma* (Gram et al., 2019), a situation in which it is individually rational for women to take as little action against domestic violence as possible, even though all women would be better off collectively if everybody invested in such action. We show that elaborations to the model based on assuming a degree of altruism in women can suggest solutions and provide new directions for research and policy.

## Background

**Fig. 1:**
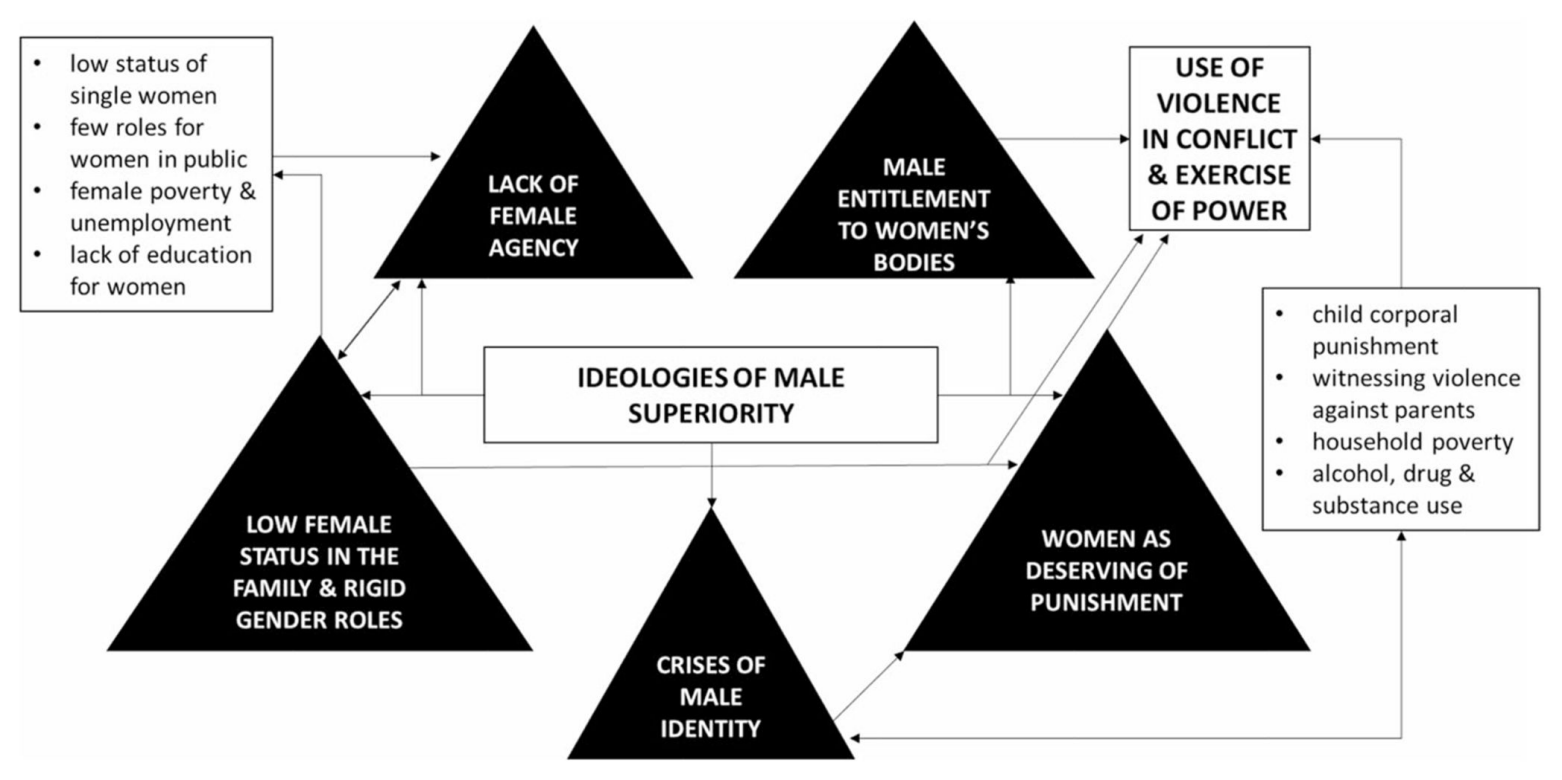
Overall framework for the determinants of domestic violence adapted from Jewkes et al. (2002). Ideologies of male superiority combine with structural and individual risk factors to promote men’s use of violence in domestic relationship.

According the socio-ecological framework (Heise, 1998), domestic violence results from the interplay of multiple factors at different levels of a social ecology: at the individual level, childhood experiences such as child abuse or marital violence between one’s own parents are major factors; at the relationship level, alcohol use, marital conflict and male control over decision-making play a role; at the community level social isolation of women and delinquent peer associations of men contribute; at the societal level, ideologies of male superiority and rigid gender roles also contribute. The framework has been adapted to centre the role of patriarchal ideology, male entitlement, and household gender roles (Jewkes et al., 2002). Figure 1 shows such an adapted framework.

Social ecological frameworks provide an overview of the complexity of domestic violence, but do not generally provide specifics as to how societal ideologies manifest at the individual level or how individual actions can reproduce ideologies. Two feminist theories, the theory of Hegemonic Masculinity (Connell, 1987) and Male Peer Support Theory (DeKeseredy and Schwartz, 2013) propose a mechanism: Men belong to a gendered power hierarchy in which they are rewarded by other men for conforming to stereotypically masculine gender roles, while men who break these norms are punished. Men are socialised into believing that gender non-conforming behaviour from female partners threatens their performance of masculine gender. This makes men use domestic violence to punish female partners for gender non-conforming behaviours. In other words, ‘social norms’, defined as expectations of informal reward or sanction from others for conforming to or deviating from a behaviour, play a key role (Hechter and Opp, 2001).

Qualitative and quantitative studies find that women’s ‘disobedience’ is cited as one of the most frequent reasons for domestic violence, whether due to neglect of household work, refusal to have sex, or arguments with the husband and in-laws (Neal and Edwards, 2017). Survey studies have found that societal acceptance of wife-beating as punishment correlates with experience of violence (García-Moreno et al., 2005; Heise and Kotsadam, 2015), while accepting attitudes among peers correlates with perpetration of violence (Mulawa et al., 2018). Other studies have found one of the most important predictors of male perpetration of violence to be the presence of male peers who explicitly verbally encourage it (DeKeseredy and Schwartz, 2013). In qualitative studies, men who engage with violence prevention programmes have encountered considerable peer resistance ranging from mockery to ostracism (Gibbs et al., 2015; Mogford et al., 2015), and fear of losing peer support has been a formidable barrier to behaviour change (Daruwalla et al., 2017).

Individual behaviour change interventions primarily seek to furnish women and men with the resources, knowledge, and skills to negotiate violence in their own relationships (Bandiera et al., 2016; Green et al., 2015; Hossain et al., 2014). These may fail to work in contexts in which pervasive social norms condone or reward male perpetrators’ use of violence to maintain control over female partners (Daruwalla et al., 2017; DeKeseredy and Schwartz, 2013). A trial of a government intervention in Papua New Guinea to prevent domestic violence through increased police presence showed no impact, as men mobilised in response to the programme to maintain power over women (Cooper, 2018). Trials of interventions to financially empower women through economic self-help groups without highly participatory group education components have generally failed to show impact on intimate partner violence (Gram et al., 2020).

Community mobilisation interventions have been developed to challenge domestic violence-related social norms through a range of activities (Abramsky et al., 2014; Pronyk et al., 2006; Wagman et al., 2015). This includes engaging police, courts, shelter homes, physical and mental health services, and non-specialist community members (Daruwalla et al., 2019). They actively involve community members in decisions about intervention delivery, including the development of strategies to tackle local priority issues (Rosato et al., 2008). Their open-ended approach makes such interventions effective, but also unpredictable (Gram, et al., 2019). For example, a documentation exercise for a communitybased intervention to prevent violence against women in Mumbai, India, found a plethora of community actions (Daruwalla et al., 2019) (Table 1). These ranged from negotiating with the family of a woman facing dowry harassment to organising a campaign to free a woman who was locked in her house and beaten by her partner. Amid such heterogeneity, common threads are hard to discern.

However, most theories of change for community mobilisation intervention emphasise community-driven attitude and social norm change as key drivers of reductions in levels of domestic violence (Gram et al., 2019). Process evaluations have evidenced the key role of ‘organised diffusion’—active, systematic efforts by community members themselves in diffusing anti-violence messages the wider community - in achieving impact (Cislaghi et al., 2019). The measurement and tracking of attitudes to violence against women is a standard component of impact evaluations (Abramsky et al., 2016; Chatterji et al., 2020; Kim et al., 2007). Social norms are increasingly monitored in high-income (Fabiano et al., 2003; Kilmartin et al., 2008) and low- and middle-income settings (Clark et al., 2018) too.

The next section formalises literature from this section into a stylised game-theoretic model^1^. Game theory is a formal theoretical framework for analysing interactive decision-making (Ferguson, 2013). A ‘game’ is any situation with multiple decisionmakers (‘players’) whose choices impact on another. Game theory predicts the behaviour of players in a setting, where the ‘payoffs’ to strategies chosen by individuals depends on strategies adopted by other individuals in the same population (McAdams et al., 2020). Game theory is widely applied in the psychological and social sciences to model trust, cooperation, and collective action (Ferguson, 2013). Public health researchers have used it to model physician prescribing behaviour in the face of rising antimicrobial resistance (Colman et al., 2019), population behaviour under voluntary vaccination (Bauch and Earn, 2004), and social distancing behaviour during COVID-19 (McAdams, 2020), but not domestic violence prevention. The following sections assume basic familiarity with game theory, but our final discussion of research and policy implications can be grasped without prior knowledge.

## Setting up the model

### Stylised assumptions

Suppose a set of women *w*_1_, …, *w_n_* live in a locality with male^2^ partners *h*_1_, …, *h_n_*, where woman *w_i_* has partner *h_i_*. We distinguish between ‘positive assumptions’, which concern the world as it is, and ‘normative assumptions’, which concern the world as it should be. For ease of reference, we have listed common variables used in mathematical formulae throughout this article in Table 2. Proofs of all theorems and propositions are available in a supplementary Technical Appendix. We make the following positive assumptions:

- Attitudes matter: men with more pro-violent attitudes are—everything being equal—more likely to perpetrate domestic violence (Abramsky et al., 2016; Flood and Pease, 2009; Fulu et al., 2013; García-Moreno et al., 2005). We assume that each male partner has attitude *a_i_*∈(−∞,∞) to violence, where higher values denote more pro-violence attitudes and lower values denote more anti-violence attitudes. Each male partner derives utility *n_ii_a_i_v_i_* from perpetrating violence at level *v_i_* for constant *n_ii_* > 0. For notational convenience, we call the payoff rate *n_ii_* so that it matches the notation below for social norm payoffs. To avoid corner cases that do not change our main argument, we assume *v_i_*∈(− ∞,∞).
- Social norms matter: men reward or punish peers for being violent. Men with more pro-violent attitudes are more likely to reward rather than punish violence in peers (Dworkin et al., 2013; Gibbs et al., 2015; Mogford et al., 2015; Peacock and Levack, 2004; Verma et al., 2006). We assume male partner *i* perpetrating violence at level *v_i_* receives norm-based reward or sanction Σ_*j*≠*i*_ *n_ij_a_j_v_i_* from other men *h_j_* for constants *n_ij_* > 0. Positive values of *n_ij_a_j_* indicate reward, while negative values indicate sanction. Positive values do not necessarily indicate reward for violence per se, but can also indicate reward for ‘enforcing discipline’ and fulfilling masculine gender roles in the household (Dworkin et al., 2013; Gibbs et al., 2015; Mogford et al., 2015; Peacock and Levack, 2004; Verma et al., 2006).
- Perpetration of violence incurs costs: perpetration of violence is not completely without psychological, social or legal cost (Bloch and Rao, 2002; Busby, 1999; Go et al., 2003; Haushofer et al., 2019). Everything else being equal, men do not prefer infinite levels of domestic violence, nor does society tolerate infinite amounts. We assume man *h_i_* perpetrating violence at level *v_i_* experiences cost $\frac{1}{2}c_{i}v_{i}^{2}$, where *c_i_* > 0 is a constant.
- Costly preventive action is possible: women taking action against domestic violence are able to change men’s attitudes, including those of their own partners (Abramsky et al., 2014; Daruwalla et al., 2019; Pronyk et al., 2006; Wagman et al., 2015), but this requires time and effort. Let *e_ij_*∈[0,∞) denote effort spent by woman *j* in changing the attitude of man *i*, with larger values denoting greater effort. Then we let *a_i_*(*e*_*i*1_, …, *e_in_*) be strictly decreasing, strictly convex functions on domain [0,∞)*^n^*. We assume, the *a_i_* are infinitely smooth functions. We model costs of effort for woman *w_j_* as $\frac{1}{2}\sum_{k}d_{kj}e_{kj}^{2}$ for constants *d_kj_* > 0. Note, efforts to change men’s attitudes do not exclusively involve women directly speaking to men and changing their minds (Cislaghi et al., 2019; Starmann et al., 2018), but can also involve women asking for support from men’s family members, asking local authorities to speak to a man, collectively protesting a man’s behaviour, or persuading couples to enter counselling programmes (Goodman and Smyth, 2011; Mannell et al., 2018).
- Violence creates suffering for survivors, but survivors themselves may tolerate it: women surviving violence suffer severe harm, whether mental, physical, sexual or economic (Devries et al., 2013; Dillon et al., 2013; Hill et al., 2016). However, survivors vary in the extent to which they perceive violence towards themselves as unacceptable or unjust (Busby, 1999; Go et al., 2003; Krause et al., 2016). We assume women experience disutility *s_i_*(1 – *t_i_*)*v_i_* from violence and level *v_i_*. *s_i_* > 0 represents the degree of suffering incurred by violence. 0 ≤ *t_i_* < 1 represents the extent to which women tolerate and view as acceptable violence in their own lives.
  Additionally, we make the following normative assumption:
- Women’s welfare should be assessed assuming zero tolerance for violence: long-term abuse is known to create ‘adaptive preferences’ in women that lead them to accept conditions of oppression (Sen, 1999). A broad consensus exists that survivors’ claims to domestic violence being justified should not be taken at face value, but evaluated in light of potential adaptive preferences (Nussbaum, 2001). As such, we evaluate woman $w_{i}^{’}\text{s}$ welfare using *W_i_*(*v_i_*, *e*_1*i*_, …, *e_ni_*) = −*s_i_v_i_* $-\frac{1}{2}\sum_{k}d_{kj}e_{kj}^{2}$, which corresponds to setting *t_i_* to 0 in her utility function.

### Game specification and solution

Let *G* be a two-stage multiplayer game with players {*w*_1_, …, *w_n_*, *h*_1_, …, *h_n_*}. Assume *n* ≥ 2. The stages of the game are as follows:

1. Each woman *w_i_* chooses a level of effort *e_ij_* in changing each man $h_{j}^{’}\text{s}$ attitudes to violence, including those of their own partner.
2. Each man *h_i_* chooses a level of violence *v_i_* to perpetrate against his partner *w_i_*.

Payoffs are awarded to men and women depending on their actions using utility functions *U_wi_*(*v_i_*, *e*_1*i*_, …, *e_ni_*) for women and *U_hi_*(*v_i_*, *e*_1*i*_, …, *e_ni_*) for men, where:

(1) $$U_{hi}=n_{ii}a_{i}\left(e_{i1},\ldots ,e_{in}\right)v_{i}+\underset{j\neq i}{\sum }n_{ij}a_{j}\left(e_{j1},\ldots ,e_{jn}\right)v_{i}-\frac{1}{2}c_{i}v_{i}^{2}$$

(2) $$U_{w_{i}}=-s_{i}\left(1-t_{i}\right)v_{i}-\frac{1}{2}\underset{k}{\sum }d_{kj}e_{kj}^{2}$$

Players make moves seeking to maximise payoffs *U_wi_* and *U_hi_*. However, women’s welfare deriving from these moves is evaluated using *W_i_* (Assumption VI). One can show:

### Proposition 1 (Nash equilibrium)

*Suppose n_ij_ and a_j_ have bounded first-order derivatives, i.e., there exists a constant M > 0 such that*$\left|\frac{\partial n_{ij}}{\partial a_{j}}\right|,\left|\frac{\partial a_{j}}{\partial e_{ji}}\right|$< *M for all i,j. Then a unique subgame perfect equilibrium exists, in which all levels of violence* $v_{j}^{*}$ *and effort* $e_{ij}^{*}$ *satisfy*:

(3) $$v_{i}^{*}=\frac{1}{c_{i}}(n_{ii}a_{i}\left(e_{i1},\ldots ,e_{in}\right)+\underset{j\neq i}{\sum }n_{ij}a_{j}\left(e_{j1},\ldots ,e_{jn}\right))$$

(4) $$e_{ji}^{*}=-\frac{s_{i}\left(1-t_{i}\right)n_{ij}}{d_{ki}c_{i}}\frac{\partial a_{j}}{\partial e_{ji}}$$

For a given set of baseline model parameters, we can represent a community that has not yet received a new policy or intervention by the resulting equilibrium levels of effort, proviolent attitude, and perpetration of violence. Changes to equilibria resulting from altering model parameters represent simulated impacts of new policies or interventions. For example, raising costs of violence might lower perpetration of violence relative to a set of baseline parameters. This would suggest that a new intervention might be able to prevent violence in the modelled community by imposing additional costs to domestic violence.

In general, parameter changes do not result in straightforward changes to equilibrium values. For example, making all women less tolerant of violence may not increase all women’s level of effort: Higher effort expended by woman *w_k_* on man *h_j_* may disincentivise woman *w_i_* from engaging with the same man, if he has already been persuaded by the efforts of *w_k_*. The net effect might be an increase in effort for some women, but a decrease for others. If the net effect of changing model parameters on women’s effort to prevent violence is ambiguous, then so might be the net effect of changing model parameters on final levels of violence. However, we can show that the latter effect is mostly unambiguous.

### Corollary 1

*Suppose*$\frac{\partial a_{j}}{\partial e_{ji}}\left(e_{j1}\ldots e_{jn}\right)=\alpha h_{ji}\left(e_{j1}\ldots e_{jn}\right)$*and*$s_{i}=\tilde{\sigma {\tilde{S}}_{i}},t_{i}={\tilde{\tau t}}_{i},\;d_{i}=\gamma {\tilde{d}}_{i},\;c_{i}=\kappa {\tilde{c}}_{i},\;n_{ij}=v{\tilde{n}}_{ij}$, *for constants*${\tilde{s}}_{i},{\tilde{t}}_{i},{\tilde{c}}_{i},{\tilde{d}}_{i},{\tilde{n}}_{ij}$, *functionsh_ji_*(*e*_*j*1_ … *e_jn_*) < 0 *and scale factors* *σ*, *τ*, *γ*, *κ*, *ν*, *α* > *0*. *Then equilibrium levels of violence decrease in σ and increase in τ and γ. The effects of changing κ and υ on levels of violence are ambiguous. Suppose for any fixed set of e_ji_*, $\frac{\partial a_{j}}{\partial \alpha }\left(e_{j1}\ldots e_{jn}\right)<0.$ *Then equilibrium levels of violence decrease in α too*.

### Social dilemmas

**Fig. 2:**
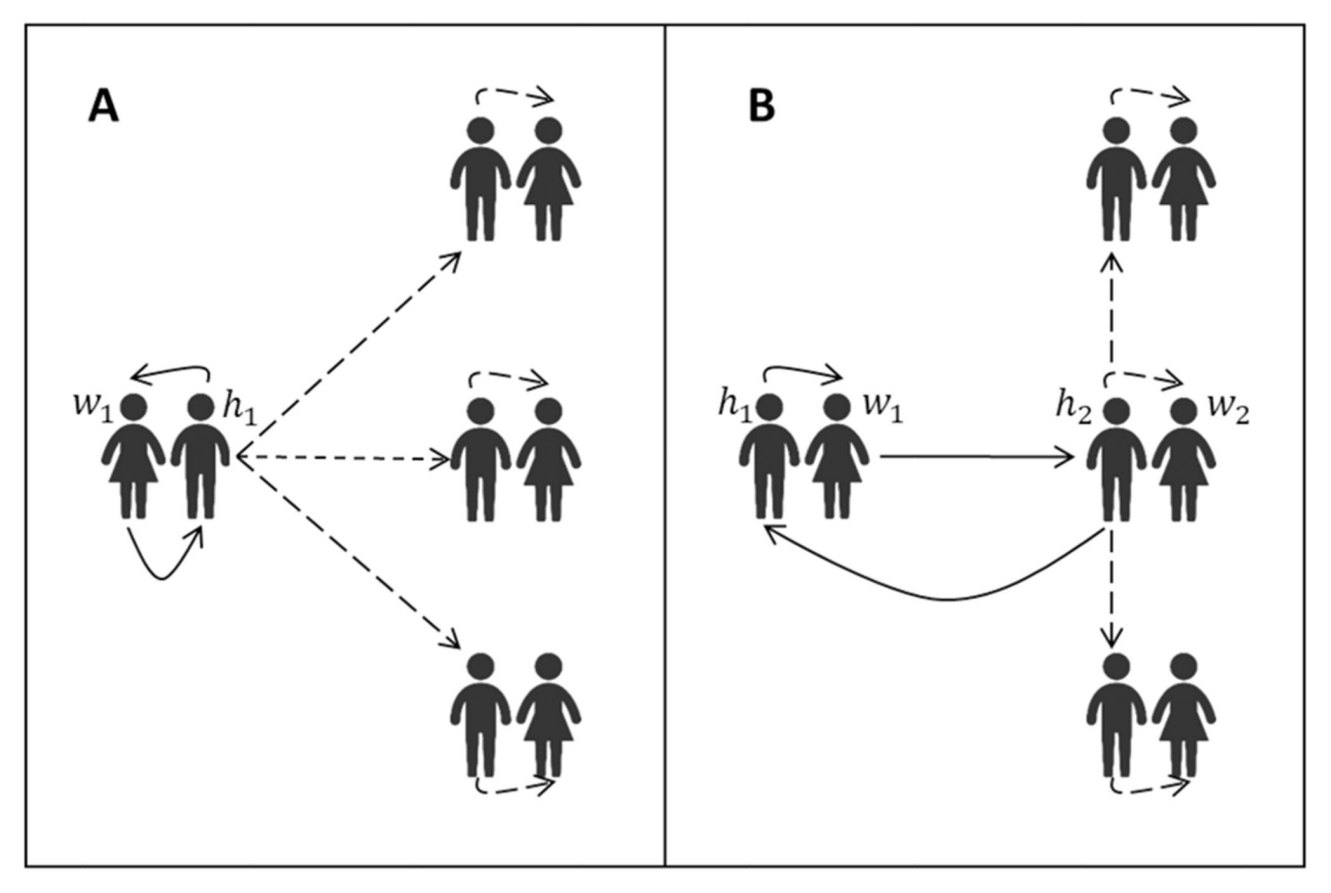
Externalities to women’s efforts to change men’s attitudes to domestic violence. **A** Woman *w*_1_’s efforts to change her partner *h*_1_’s attitudes to violence affect her welfare, because he becomes less likely to perpetrate domestic violence (solid lines), but this produces externalities for other women (dotted lines). **B** Woman *w*_1_’s efforts to change neighbouring man *h*_2_’s attitudes to violence affect her welfare, because *h*_2_ will be less willing to encourage *h*_1_ to perpetrate domestic violence (solid lines). However, her efforts produce externalities for other women’s relationships, including the relationship between *h*_2_ and *w*_2_ (dotted lines).

Women’s efforts to change the attitudes of their own or neighbouring partners produce benefits for other women’s relationships that they do not themselves directly experience (Fig. 2). These benefits are conventionally called ‘positive externalities’ (Cornes and Sandler, 1996). Women may find it individually rational to abstain from taking preventive action, even though they would be better off if all women collectively agreed to act, a situation called a ‘social dilemma’ (Gram et al., 2019). We can prove that a subset of women always exists for whom increasing levels of effort to prevent violence beyond equilibrium levels would improve their individual welfare:

### Theorem 1 (Social dilemmas)

*Suppose two women w_1_ and w_2_ have equilibrium levels of effort*$e_{i1}^{*}$*and*$e_{j2}^{*}$*with regard to influencing the attitude of men h_i_ and h_j_ for some*,*j* ∈ 1, …, *n*. *Then there exist δe_i1_*,*δe_j2_ > 0 such that the welfare of w_1_ and w_2_, W_1_ and W_2_, would be greater if they simultaneously exerted effort levels* $e_{i1}^{*}+\delta e_{i1}\;and\;e_{j2}^{*}+\delta e_{j2}$ *respectively compared to* $e_{i1}^{*}\;and\;e_{j2}^{*}.$

The social dilemma is likely to be particularly acute when the community is large (*n* >> 1) and social norms are only weakly responsive to individual women’s efforts to change them $(\frac{\partial n_{ji}}{\partial a_{i}}\to 0$ for *i* ≠ *j*). Such a situation approximates classic collective action problems studied in economics and political science (Olson, 1971). Here, the impact of an individual woman trying to persuade men to become less violent is negligible, even though significant reductions in violence could be obtained were all women in the community to contribute simultaneously. One woman investing huge effort to change men’s attitudes would have little impact relative to many contributing small amounts each.

### Scenario 1

We illustrate our results using examples. Interventions that make it easier for women to take preventive action—i.e., reduce the costs of effort or increase the effectiveness of effort—may seem like obvious solutions to inaction at equilibrium. However, Theorem 1 shows that such interventions do not eliminate the presence of social dilemmas as they do not change the relationship between individual and collective rationality. This limits their impact on violence and women’s welfare: if individual benefits to action are almost zero relative to cost, it might require unrealistically large changes in model parameters to generate impact.

Let us model a scenario where the social dilemma of preventing violence is severe: social norms encouraging domestic violence are widespread and influential relative to women’s ability to change men’s attitudes to violence. We parameterise attitudes as a linear function of efforts *a_j_*(*e*_*j*1_, …, *e_jn_*) = 2−∑*_i_f_ji_e_ij_* and set *f_ji_* = 0.1 to indicate a weak ability to alter men’s attitudes. We set the number of couples *n* = 10 to model a small community and *t_i_* = 0.5 to indicate a degree of tolerance for violence among women. We set *s_i_* = *c_i_* = *n_ji_* = *d_ji_* = 1. As all couples have identical parameters, all women experience the same level of violence, exert the same level of effort and experience the same level of welfare; we can also consider any plotted values to be the values of a ‘representative average woman’.

**Fig. 3:**
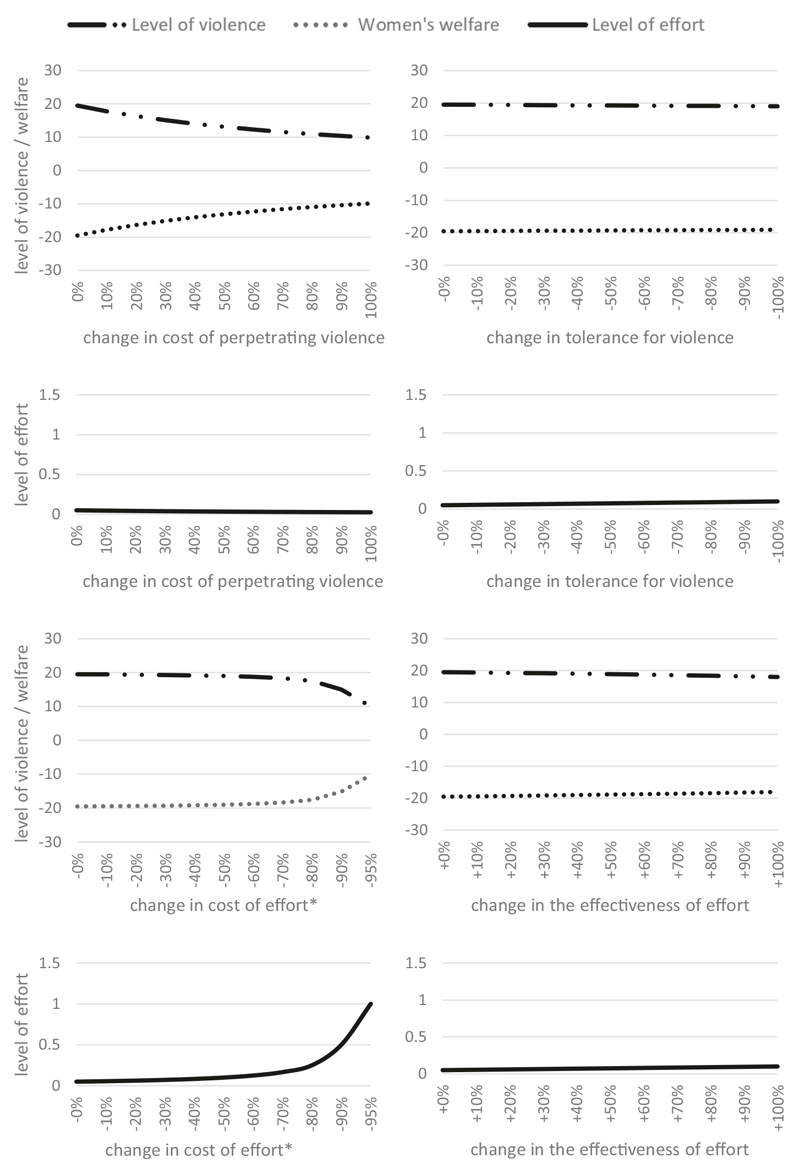
Simulated impacts of changes to model parameters on equilibrium outcomes (Scenario 1). Only increased cost of violence perpetration materially improves women’s welfare for moderate-sized parameter changes. *Cost of effort must be strictly positive, so the last data point is −95% instead of −100%.

Figure 3 shows the effect of varying simulated parameters^3^ on equilibrium levels of violence, effort, and welfare. Interventions that reduce women’s tolerance for experiencing violence or increase their effectiveness at changing men’s attitudes have almost no discernible impact on any outcome. Interventions to reduce the cost of effort only begin to have material impact when the cost has been reduced to almost zero. Interventions to increase costs of perpetrating violence do have an impact, but this impact is independent of women’s efforts to prevent violence, which stay near constant throughout. These results show the complexity of mobilising collective action to prevent domestic violence.

### Scenario 2

Let us consider an alternative scenario. Corollary 1 indicates that increases in the cost of perpetrating violence have ambiguous effects. Counterintuitively, it is theoretically possible for more severe punishment for violence to exacerbate levels of violence. In our model, this effect arises from women themselves anticipating the deterrent effects of externally imposed punishment, which leads women to reduce effort in changing men’s attitudes to violence, thus weakening or nullifying any possible deterrent effect of external punishment.

We can simulate this perverse effect with a slight modification to Scenario 1. As before, we parameterise attitudes as a linear function of efforts *a_j_*(*e*_*j*1_, …, *e_jn_*) = 2−∑_*i*_ *f_ji_e_ji_*. As before, we set the effectiveness of attitude change to *f_ji_* = 0.1, tolerance for violence to *t_i_* = 0.5 and cost of preventive effort to *d_ji_* = 1. However, we now increase the strength of norm-based reward and punishment to *n_ji_* = 5, the number of couples to *n* = 20, and the degree of suffering incurred by violence to *s* = 1.5. We set the cost of perpetrating violence to *c_i_* = 0.4 and then look at the effect of gradually increasing it.

**Fig. 4:**
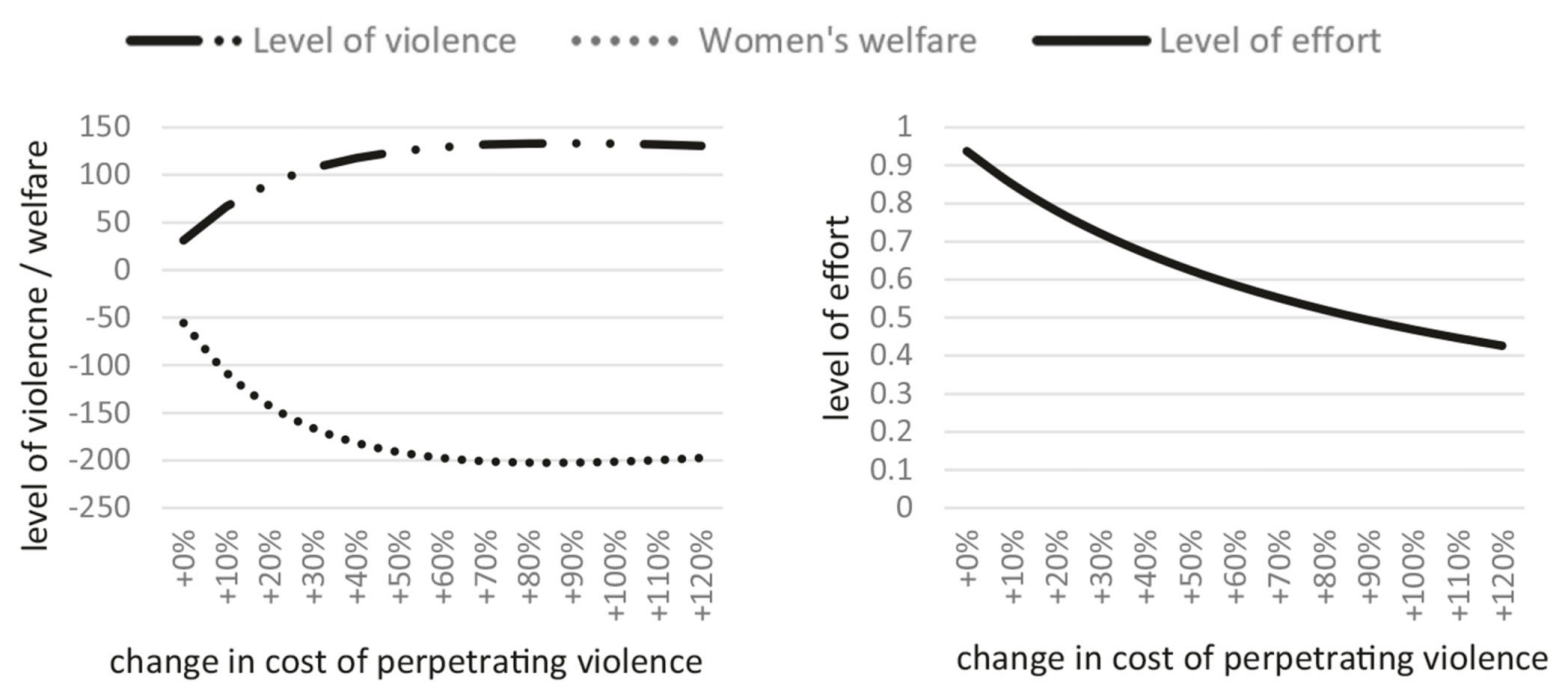
Simulated impacts of increases in the cost of violence perpetration on equilibrium outcomes (Scenario 2). Moderate-sized increases in cost of perpetrating violence can result in *greater* levels of violence.

Figure 4 shows the result. We see that increases in the cost of violence initially result in greater levels of violence before slowly reducing in intensity after the cost of violence has more than doubled. In contexts with virtually no external sanction for violence, women’s own efforts at attitude change are the primary force keeping violence at bay. Increasing costs of violence at a point where external sanction is still weak can lead women to overly relax their own preventive efforts, which results in the observed rise in violence.

## Modelling solutions

Given the inherent challenges in motivating collective action to prevent violence in the face of social dilemmas, it is important to consider possible solutions. As discussed above, community mobilisation interventions have achieved varied success in reducing violence (Abramsky et al., 2014; Chatterji et al., 2020). Differences in impact reflect differences in context, implementation, and time allotted to the intervention (Gram et al., 2019). One indicator of quality of implementation may be the ability of such interventions to encourage community participation beyond baseline levels. Indeed, if no social dilemma were present, one would need to explain why community members had not already organised effective collective action prior to the introduction of an external intervention.

Conventional economic solutions to social dilemmas based on direct material incentives—tax credits, financial subsidies, property rights, or legal contracts (Oliver, 1993; Olson, 1971)—are largely infeasible. Financial rewards for laywomen to challenge patriarchal attitudes among their neighbours are unlikely to work if men know that women have adopted feminist values simply because they were paid to do so. Solutions based on property rights and legal contracts are also unworkable when the externalities in question—reductions in domestic violence due to changed social norms—are difficult-to-observe, intangible goods.

Instead, we might consider solutions based on altruism.

### Process-based altruism

Process-based altruism refers to an intrinsic motivation to take action irrespective of any instrumental benefit (Kyriacou, 2010). This can be due to righteous anger, a sense of injustice, moral duty, or principle (Schuessler, 2000; Smith et al., 2012). It could also be due to a sense of empowerment or ability to lead on a moral issue. We can model process-based altruism by adding an intrinsic benefit term to women’s utility Eq. (2). Let

(5) $$U_{wi}=\underset{k}{\sum }be_{ki}-s_{i}\left(1-t_{i}\right)v_{i}-\frac{1}{2}\underset{k}{\sum }d_{ki}e_{ki}^{2}$$

for constant *b* >0. ∑*_k_be_ki_* only depends on women’s effort, not their experience of violence. This avoids incentive incompatibilities caused by women’s motivation for effort to prevent violence being conditional on such effort directly benefiting themselves and their own relationship to their partner. However, as motivation to take action is no longer tied to expectations about impact on violence, this mechanism might also theoretically result in over-investment in action against violence. Women might take action at great personal cost with little impact on violence, because ‘doing something is better than nothing’. Finding the optimal value for *b* is nontrivial, but we might obtain insight by considering the following:

### Proposition 2

*Women’s welfare is increasing in b if and only if for all i*:

(6) $$b\underset{k}{\sum }d_{ki}\frac{\partial e_{ki}^{*}}{\partial b}<-\underset{k}{\sum }\underset{j}{\sum }\tau_{ji}\frac{s_{i}n_{jk}}{c_{i}}\frac{\partial a_{k}}{\partial e_{kj}}\frac{\partial e_{kj}^{*}}{\partial b}$$

where $\tau_{ji}=\{\begin{array}{c} t_{i}\;if\;j=i\\ 1\;if\;j\neq i \end{array}$

Proposition 2 states that we can always improve woman $w_{i}^{’}\text{s}$ welfare by promoting greater intrinsic motivation to act among all women, as long as her marginal cost of effort is outweighed by additional benefit received from other women becoming more motivated to act. This suggests *b* can be large without causing over-investment in the context of social dilemmas, where the marginal benefit of individual effort is negligible, but that of collective effort is substantial.

### Empathetic altruism

We use ‘empathetic altruism’ to refer to a motivation to help people in need caused by feelings of concern, sympathy, compassion or identification with their pain (Penner et al., 2005). This differs from process-based altruism, which refers to a desire to act due to the perceived intrinsic goodness of such action irrespective of its consequences for oneself or others. We can model empathetic altruism by adding other women’s suffering to women’s utility function^4^:

(7) $$U_{w_{i}}=-s_{i}\left(1-t_{i}\right)v_{i}-\eta \underset{j\neq i}{\sum }s_{j}v_{j}-\frac{1}{2}\underset{k}{\sum }d_{ki}e_{ki}^{2}$$

where 0 ≤ *η* represents weight given to other women’s suffering. *η* is upwardly unbounded, so a woman might put larger weight on others′ suffering than on her own. Women can now account for the positive externalities of their own actions when deciding on effort levels. As with process-based altruism, this mechanism may increase effort levels toward optimality, but may also overshoot and compel women to expend costly effort without adequate compensation to themselves for their own involvement in collective action.

### Proposition 3

*Women’s welfare is increasing in η if and only if for all i*:

(8) $$-\eta \underset{k}{\sum }\underset{j\neq i}{\sum }\frac{s_{j}n_{jk}}{c_{j}}\frac{\partial a_{k}}{\partial e_{ki}}\frac{\partial e_{ki}^{*}}{\partial \eta }<-\underset{k}{\sum }\underset{j}{\sum }\tau_{ji}\frac{s_{i}n_{ik}}{c_{i}}\frac{\partial a_{k}}{\partial e_{kj}}\frac{\partial e_{kj}^{*}}{\partial \eta }$$

$\text{where}\tau_{ji}=\{\begin{array}{c} t_{i}\;if\;j=i\\ 1\;ifj\;\neq i \end{array}.$

Proposition 3 states that increasing *η* improves women’s welfare, as long as the degree of benefit produced by each woman for neighbouring women is outweighed the degree of benefit received by that woman from the actions of neighbouring women. This suggests that empathetic altruism works best in homogeneous populations where flows of benefit between women are more likely to be equal in size.

### Reciprocal altruism

Reciprocal altruism5 refers to a motivation to provide favours for other individuals in anticipation of being able to receive future favours in return (Trivers, 2006). It differs from empathetic altruism in that individuals help others in proportion to the degree they expect others to help them in return, rather than the degree to which they expect others’ suffering to be alleviated by such help. It differs from process-based altruism in that individuals do take the consequences of their actions into account in deciding whether to act. We model reciprocal altruism by assuming women are motivated to match the effort levels of other women (Gächter, 2006):

(9) $$U_{w_{i}}=-s_{i}\left(1-t_{i}\right)v_{i}+\rho \underset{k}{\sum }\underset{j\neq i}{\sum }e_{ki}e_{kj}+\frac{1}{2}\rho \underset{k}{\sum }e_{ki}^{2}-\frac{1}{2}\underset{k}{\sum }d_{ki}e_{ki}^{2}$$

0 ≤ *ρ* represents weight given to matching other women’s efforts with higher values indicating greater willingness to match^6^. We can show:

### Proposition 4

*Suppose*$0\leq \rho <\frac{1}{n}.$*Women’s welfare increases in ρ if and only if for all i*:

(10) $$-\frac{\rho }{1-n\rho }\underset{k}{\sum }\underset{j}{\sum }\frac{s_{j}\left(1-t_{j}\right)n_{jk}}{c_{j}}\frac{\partial a_{k}}{\partial e_{kj}}\frac{\partial e_{ki}^{*}}{\partial \rho }<-\underset{k}{\sum }\underset{j}{\sum }\tau_{ji}\frac{s_{i}n_{ik}}{c_{i}}\frac{\partial a_{k}}{\partial e_{kj}}\frac{\partial e_{kj}^{*}}{\partial \rho }$$

$\text{where}\;\tau_{ji}=\{\begin{array}{c} t_{i}\;if\;j=i\\ 1\;if\;j\neq i \end{array}.\text{for}\rho \geq \frac{1}{n},$ a subgame perfect equili brium may not exist.

Proposition 4 shows that the marginal cost of increasing *ρ* may rise rapidly, as women’s commitment to match each other’s investments in collective action creates mutually reinforcing feedback loops: $\frac{\rho }{1-n\rho }\to \infty \;\mathrm{as}\;\rho \to \frac{1}{n}$ from the left. This suggests that even small amounts of reciprocity can entail large commitments in effort.

### Scenario 3

Let us now consider the impact of increasing altruism on women’s outcomes. For simplicity, we use the same functional forms and initial values that we used in Scenario 1. Recall that all couples have identical parameters, so all women experience the same level of violence, exert the same level of effort and experience the same level of welfare, i.e., we can consider values plotted to concern a representative ‘average woman’.

**Fig. 5:**
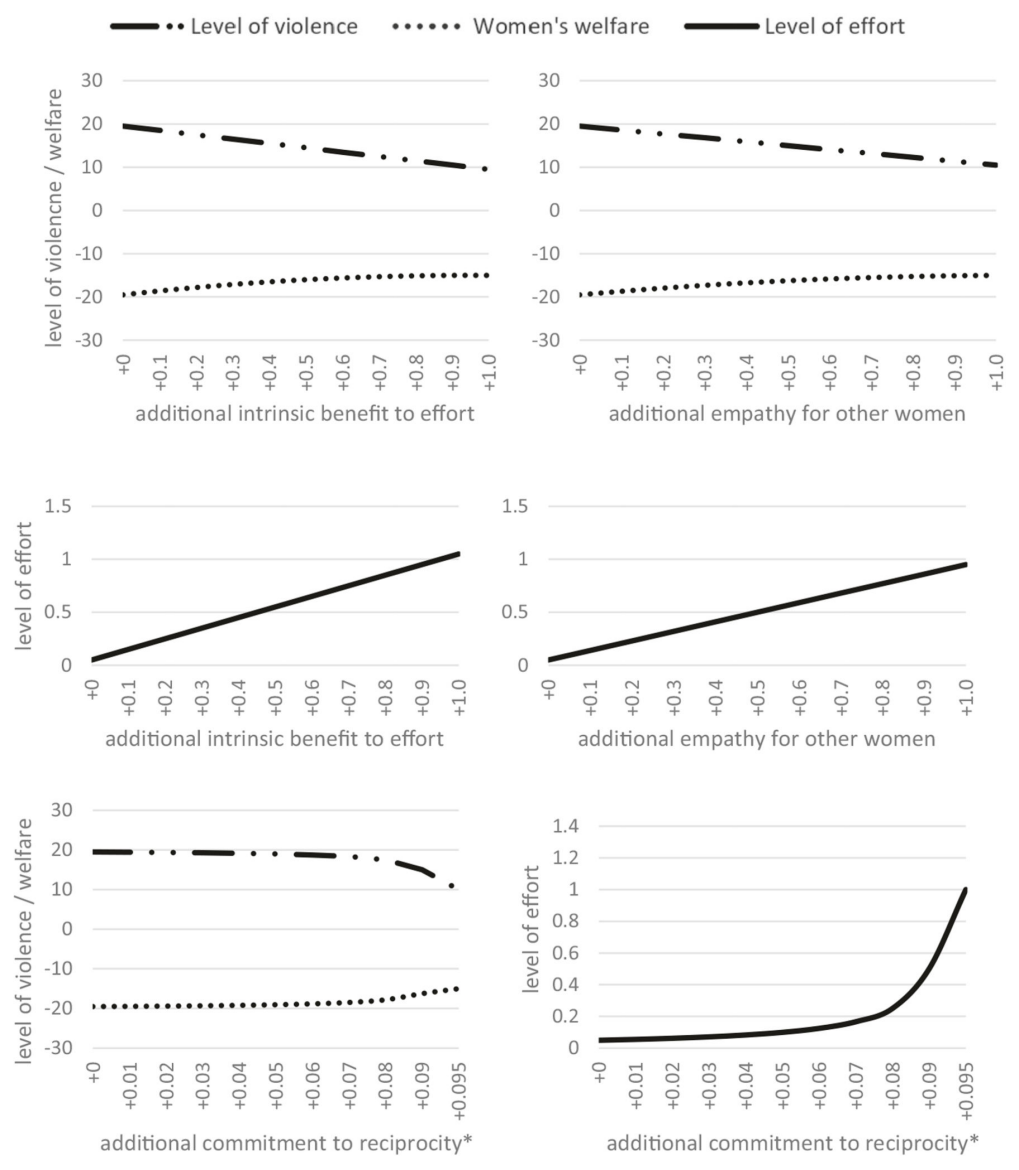
Simulated impacts of changes to women’s level of altruism on equilibrium outcomes (Scenario 3). Increases in intrinsic benefit, empathy and reciprocity reduce levels of violence and increase women’s welfare. *No equilibrium exists for reciprocity level *ρ* = 0.1, so the last data point is +0.095, not +0.1.

Figure 5 shows the simulated effect of increasing process-based, empathetic and reciprocal altruism on equilibrium levels of violence, effort to prevent violence, and women’s welfare. In contrast to Scenario 1, we find an immediate increase in women’s effort levels and a concomitant decrease in levels of violence in response to even small amounts of altruism. For example, an increase in average levels of effort from zero to one unit results in a halving of experienced violence. If a woman wanted to achieve comparable improvements to her own relationship through her own efforts alone—i.e., by investing all her effort in changing her own male partner’s attitudes without involving other women—it would require nearly a hundred-fold greater level of effort.

We found little difference in our simulated scenario between the effects of process-based and empathetic altruism. However, reciprocal altruism had little effect on effort and violence levels until the degree of reciprocity neared $\frac{1}{n},$ when levels of effort changed rapidly, as predicted by Proposition 4.

### Scenario 4

**Fig. 6:**
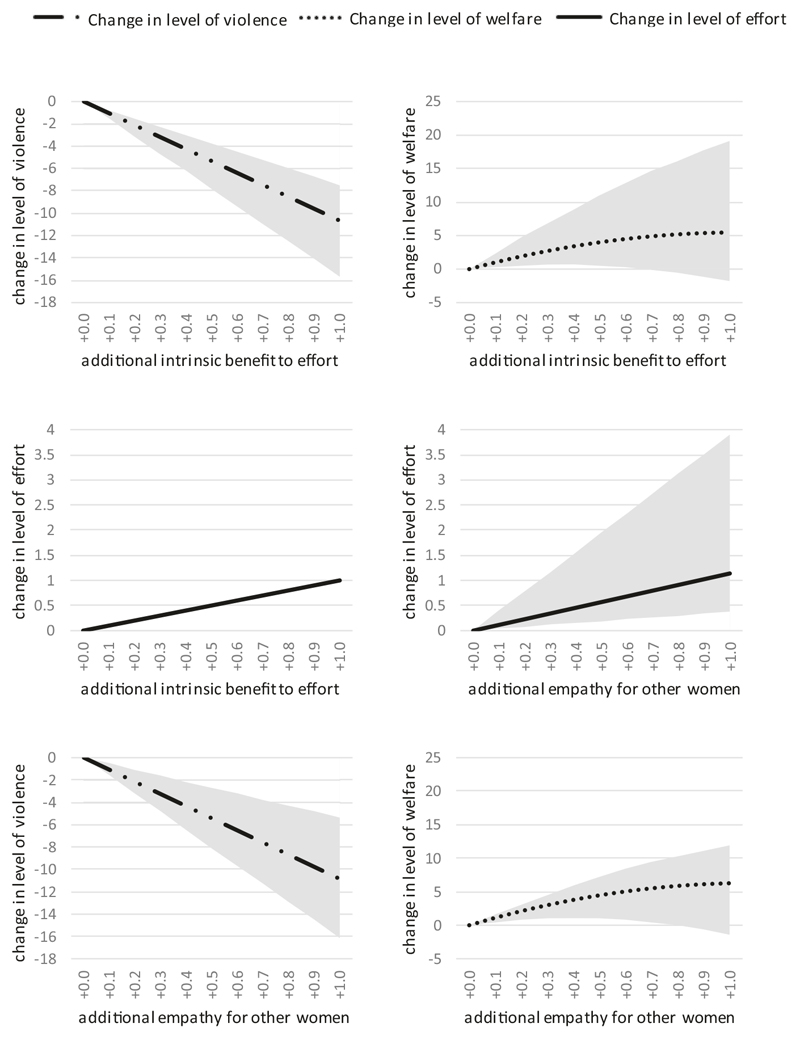
Simulated impacts of changes to women’s altruism in the presence of heterogeneity between individual couples (Scenario 4). Heterogeneity in model parameters for individual couples create heterogeneity in outcomes. Lines represent median changes among all women. Grey bands indicate the full range of changes among all women.

Let us now consider the effect of introducing heterogeneity between couples. We begin with the ‘representative woman’ model in Scenario 1 and then vary the parameter values for each couple by an independent random amount ranging from a threefold reduction to a threefold increase (see Supplementary Appendix for further details). We next gradually vary levels of altruism and inspect their effect on violence-related outcomes. The minimum, median, and maximum change in such outcomes in response to changes in levels of altruism is plotted in Fig. 6. We have not displayed the effect of changing women’s reciprocal altruism, as it offers essentially the same lessons as the other two forms of altruism.

The median changes in violence-related outcomes mirror results from Scenario 3. The median drop in violence after increasing intrinsic benefit by +1.0 is still approximately ten units. The median change in welfare is still approximately five units. The median increase in effort is the same as before^7^. However, there is considerable variation between individual couples. Long before the welfare of the median woman peaks, the welfare of the minimum woman peaks. If all women experienced +1.0 units greater process-based altruism, the median and maximum woman would experience improved welfare, but the minimum woman would be worse off compared to the situation where no altruism existed. The results for empathetic altruism are similar.

## Implications

Community mobilisation researchers have long asserted that coordinated, collective action is necessary to prevent domestic violence, but have not fully explained why this is the case (Gram et al., 2019). One of the main benefits of explicit mathematical models is an enhanced ability to interrogate the assumptions behind our theories. We show that, under Assumptions I-VI, women’s collective action to prevent domestic violence becomes a social dilemma, which requires coordinated action to overcome. Should any one of the assumptions fail to hold, social dilemmas may not exist and interventions to address domestic violence may not need to involve collective action at all. Should they hold, community mobilisation researchers might need to re-interrogate existing ideas of how to best encourage collective action.

### Not all contexts call for collective action to prevent violence

Assumptions I and II in our model require the existence of social norms that impose sanctions or rewards on perpetrators of violence. Without these assumptions, our model does not predict externalities or social dilemmas to arise. Domestic violence-related social norms have been documented in contexts in which community members live in close geographic and social proximity such as college campuses in the United States (DeKeseredy and Schwartz, 2013), urban slums in India (Verma et al., 2006) and Uganda (Kyegombe et al., 2014), social clubs in Tanzania (Mulawa et al., 2018), or rural communities in Kenya (Hatcher et al., 2013). For example, residents of Mumbai slums generally keep their door open for ventilation and socialisation given the small amount of space available to them inside (Gram et al., 2020). Under such conditions, residents generally felt they would immediately notice if anyone closed their door and perpetrated domestic violence. Studies of violence prevention programmes in Kenya (Haushofer et al., 2019) and Mexico (Bobonis et al., 2015) found reductions in domestic violence within one household was accompanied by decreases in violence among neighbouring households.

However, contexts in which such social norms are weak or non-existent do not obviously present risks of social dilemmas. For example, rural US households have been argued to be socially isolated due to large physical distances between homes and a culture of silence surrounding household affairs (Anderson et al., 2014). A social network analysis of rural households in Honduras found attitudes to violence were almost exclusively determined by household members rather than community members (Shakya et al., 2016). Men and women in Beijing, Seoul, and Hong Kong have been found to keep domestic violence secret from their closest friends and relatives for fear of losing face (Bouhours and Broadhurst, 2015; Emery and Wu, 2020). In these contexts, it may be challenging for men to reward or punish other men for perpetrating domestic violence or enforcing gender normative behaviour in their partners, as they do not have access to such information.^8^

If social dilemmas do not exist, it is unclear to what extent implementers are justified in pushing for higher levels of collective action beyond individually rational levels, as women living in such contexts do not face significant returns to coordinated action. Extensive participation might take time and energy away from activities that women themselves genuinely value without proportionate reductions in their experience of violence. Collective action-oriented community mobilisation might be inefficient in such cases relative to individual interventions such as couples counselling (Stern and Niyibizi, 2018) or economic empowerment (Green et al., 2015).

### Conventional solutions to prevent violence may not work

Surprisingly, our model suggests that a number of conventional techniques for preventing violence in community mobilisation interventions may not always work. Theorem 1 showed that changes to women’s tolerance for experience violence do not eliminate the presence of social dilemmas. Scenario 1 showed that lower tolerance for violence may matter to neither women’s decision to take action nor men’s decisions to perpetrate violence. Intervention designers should not automatically expect consciousness-raising efforts encouraging women to reject violence (Watts and Hipolito-Delgado, 2015) to lead to collective action.

Similarly, Scenario 1 showed that changes to the cost of women’s preventive action or the effectiveness with which they changed men’s attitudes might not materially alter women’s level of participation in collective action or men’s level of perpetration of violence. Theorem 1 showed that such changes do not remove the presence of social dilemmas. Interventions that make it easier, less risky or less time-consuming for women to take action against domestic violence should not always be expected to stimulate significant collective action.

Scenario 2 even showed that increasing the cost of violence perpetration can backfire and cause greater levels of violence, if women anticipate its deterrent effect and invest less effort in prevention as a result. This mirrors models of criminal punishment, which predict null effects of harsher penalties on crime because police officers anticipate their deterrent effect and relax law enforcement efforts as a result (Tsebelis, 1990). Behavioural evidence partially bears this out by showing that higher punishments do indeed lead to relaxed efforts to control crime in lab experimental settings (Rauhut, 2009, 2015). However, caution is needed before extrapolating from these findings, as no field experiments have been done.

We proposed solutions based on process-based, empathetic, and reciprocal altruism, with roots in the literature on social movements and collective action. Scenarios 3 and 4 showed that such mechanisms could stimulate action and reduce violence. Studies of feminist social movements have documented the importance of emotions in activating process-based altruism towards other women, particularly righteous anger arising from awareness of patriarchal oppression (Hercus, 1999). Collective identity has been found to foster empathetic altruism, as women’s understanding of themselves as a social group makes them feel that ‘violence against one woman constitutes automatically also a threat against… others’ (Kreft, 2019). Researchers studying sexual assault in the US have evidenced the role of empathy in promoting bystander action (Beeble et al., 2008; Yule et al., 2020). Finally, reciprocal altruism has been observed in contexts where women already had reciprocal economic ties, such as microcredit groups in which women mobilised to support other group members against domestic violence (Sanyal, 2009).

### Valuing the benefits of community participation is nontrivial

Cost-effectiveness analyses of community mobilisation interventions to prevent domestic violence have hitherto valued the cost of time for community volunteers as a linear function of local wage rates, while equating benefits with the total number of cases averted by the whole intervention (Jan et al., 2011; Michaels-Igbokwe et al., 2016). A counterfactual analysis of the causal impact on domestic violence of social norms-based interventions in Uganda explicitly avoided accounting for non-linear returns to action (Kadengye et al., 2019). However, simply dividing the total number of cases averted by the total time spent on intervention activities provides an incomplete picture of the relationship between benefits and costs, when there are externalities to time spent on intervention activities. Time spent by women engaging with a community mobilisation intervention can produce benefits, not only for themselves, but also for other women in the community through social norm change. Our analysis highlights the need to identify and measure externalities when costing community mobilisation interventions (Brouwer, 2019).

Researchers have justified lack of collective action in community mobilisation interventions with reference to women’s verbal statements about lacking time and energy to invest (Hargreaves et al., 2009). Bystander action researchers have attributed inaction in the face of violence to fears of getting hurt, causing embarrassment, or being targeted by gossip for intervening in “others’ business” (Banyard, 2011). Community members may also believe that getting involved achieves little or even harms survivors of violence (Latta and Goodman, 2011). Yet, if women are stakeholders in a social dilemma, it is simultaneously true that (1) women are individually justified in abstaining from action, because it is not worth the time, costs and risks involved and (2) women are denying themselves benefits that more than outweigh the time, costs and risks involved by collectively abstaining. This moral aspect of social dilemmas has been debated in research on environmental (Hourdequin, 2010) and feminist (Slote, 2007) ethics, but not in community mobilisation.

## Discussion

By modelling collective action to address domestic violence, we walk the less trodden path. Previous mathematical models of domestic violence have focused on household-level determinants of violent behaviour, modelling domestic violence as a function of intra-household bargaining power (Bloch and Rao, 2002; Eswaran and Malhotra, 2011; Farmer and Tiefenthaler, 1997; Haushofer et al., 2019; Tauchen et al., 1991). In such models, men perpetrate violence due to intrinsic preferences for violence or for the sake of extracting resources from their female partners. Women can only prevent domestic violence by leaving the relationship, strengthening their bargaining power or acquiescing to their male partners’ demands for resources. Other models have examined intergenerational impacts on domestic violence of witnessing violence between parents as a child (Koç and Erkin, 2012; Pollak, 2004) or modelled women’s choice to report violent partners to the police (Aizer and Dal Bo, 2009). None of these modelled women’s collective efforts to change social norms.

Like all models, our model relied on assumptions for deriving predictions and explanations of behaviour. Most fundamentally, we modelled individual decision-making as a process of rational utility-maximisation. We believe this assumption is more realistic than models in which individuals act reflexively without considering costs or benefits of action (Drigo et al., 2012; Rigby and Johnson, 2017). Our assumption is consistent with evidence on social mobilisation (Rogers et al., 2018), prosocial (Bierhoff, 2005), and help-seeking (Fugate et al., 2005) behaviour, which demonstrates that individuals are sensitive to costs and benefits of such behaviour. In contrast to strict rational choice models, we included significant social and psychological components. In particular, we modelled men’s preferences for violence as being amenable to change through the sustained efforts of women in their community. This aligns with recent assessments of the evidence base in economics calling for a move towards a view of individuals as ‘enculturated actors’ whose preferences are subject to social influences (Hoff and Stiglitz, 2016).

Nonetheless, our model has limitations. First, we only modelled women’s participation in collective action. Men’s participation in action to prevent violence against women does not straightforwardly entail the presence of social dilemmas as they do not directly benefit from reduced violence; indeed, active male engagement in violence prevention has been weaker compared to female engagement in programmes involving both genders^9^ (Chakraborty et al., 2018; Hatcher et al., 2020; Jejeebhoy and Santhya, 2018). Second, our model focused on the interaction between attitudes, social norms, and behaviour, but did not explicitly model beliefs. Models of efficacy beliefs (Lemoine, 2019) can assess the impact of women under- or over-estimating their actual influence over men’s attitudes. Women may for example refrain from action because they lack confidence in their own strength. Models of ‘pluralistic ignorance’ (Centola et al., 2005), in which individuals over- or under-estimate support for a social norm, may be needed if such misperceptions prevent collective action. We did not incorporate belief updating to avoid adding complexity to an already-complex model.

## Conclusion

We integrated widely known notions of patriarchal gender norms, male peer support, and hegemonic masculinity from the literature on violence against women into a mathematical model of social change through community mobilisation. We showed that, under a broad range of conditions, women’s decision to participate in collective action to prevent domestic violence becomes a social dilemma. In such a situation, our model predicts process-based, empathetic, and reciprocal altruism to work in mobilising collective action and reducing levels of domestic violence. Our model provides a basis for developing of theories of violence prevention that employ mathematical modelling to explore the complex system dynamics of collective action. Future research might look for predicted relationships between altruism and collective action. Such work would benefit from collaborative work with game theorists, gender scholars, and public health researchers. Interdisciplinary studies combining a solid theoretical basis with empirical investigation should trial such solutions in an effort to improve violence prevention work.

## Supplementary Material

**Supplementary information** The online version contains supplementary material available at https://doi.org/10.1057/s41599-021-00730-z.

Appendix

## Figures and Tables

**Table 1:** Actions taken by community members to address violence against women in a community-based intervention in Mumbai, India. Adapted from (Daruwalla et al., 2019)

Incident and action	Form of violence
A local volunteer helped a bride-to-be deal with dowry demands from the groom’s family which risked escalating into violence. The volunteer explained the law and her rights to the bride-to-be and discussed these with her family as well.	Domestic violence
A local volunteer intervened when a couple were fighting in the street, accompanied by their two young children. They had doused themselves in kerosene and were threatening to set fire to themselves. A volunteer talked them out of their plan, took their matchsticks away from them and called the police. The couple returned later to thank the volunteer.	Domestic violence
A woman fled to her mother’s home after her partner hit her, but he came after her and attacked both her and her mother. They shouted for help and members of the local woman’s group detained the man and called the police, who arrested him. The women persuaded the couple enter into a counselling programme.	Domestic violence
A woman told a community organiser that her neighbour was being beaten and locked in the house by her partner. The community organiser organised campaigns in the area and gathered a group who visited the house repeatedly, heard the woman inside, and got the police to effect entry. The local women and the community organiser persuaded the woman and her partner to enter into a counselling programme.	Domestic violence
A local volunteer heard her neighbour’s 7-year-old daughter shouting for help when a local man attempted to rape her. She gained access to the house, prevented him from leaving, and called for help. A group of neighbours took him to the police station, where he was arrested.	Child sexual abuse
A local volunteer led a women’s group to repeatedly confront a group of drug users who were harassing women. The harassment stopped and the volunteer became a community leader in a male-dominated area.	Sexual harassment
A young married woman was being harassed by two gang members. When she rejected their advances, they beat her up, set her on fire and locked her in her house. At the public mourning after her death, a large group of relatives and friends banded together and communicated with the police to ensure due process. The perpetrators were jailed.	Assault and murder

**Table 2:** List of variables Identifiers refer to specific agents in the world, that is men and women. Parameters refer to fixed model parameters. State variables are states of the world that may change due to agents’ choices.

Variables	Range	Type	Description
*n*	0,1,2,3,…	Parameter	Total number of couples
*w_i_*	N/A	Identifier	Woman number *i*, partner to man *i*
*h_i_*	N/A	Identifier	Man number *i*, partner to woman *i*
*a_i_*	(−∞,∞)	State variable	Attitude to violence of man *i*
*e_ij_*	[0,∞)	State variable	Effort of woman *j* to change man *i*
*n_ij_*	(0,∞)	Parameter	For *i* ≠ *j*: Norm-based payoffs imposed on man *i* by man *j* in response to violence perpetrated by man *i*
			For *i* = *j*: Intrinsic payoffs experienced by man *i* in response to violence perpetrated by himself
*v_i_*	(−∞,∞)	State variable	Level of violence perpetrated by man *i*
*c_i_*	(0,∞)	Parameter	Marginal cost of violence to man *i*
*d_ij_*	(0,∞)	Parameter	Marginal cost of effort by woman *j* to change man *i*
*s_i_*	(0,∞)	Parameter	Rate of suffering incurred by violence to woman *i*
*t_i_*	[0,1]	Parameter	Tolerance for violence expressed by woman *i*
*U_wi_*	(−∞,∞)	State variable	Utility level of woman *i*
*U_hi_*	(−∞,∞)	State variable	Utility level of man *i*
*W_i_*	(−∞,∞)	State variable	Level of welfare of woman *i*
*b*	(0,∞)	Parameter	Intrinsic motivation to take action against violence
*η*	(0,∞)	Parameter	Rate of empathy towards other women’s suffering
*ρ*	(0,∞)	Parameter	Degree of reciprocity in women’s investment of effort

## Data Availability

All data generated or analysed during this study are included in this published article.

## Footnotes

1 Note, we are not claiming that our model captures the full complexity of preventing domestic violence through community mobilisation. Rather, we are putting a magnifying glass to social interactions between community members in order to analyse the contribution of grassroots collective action to the prevention of domestic violence in the context of a community mobilisation intervention.

2 For simplicity, we describe the agents in our model as women and men living together as couples, for example as husband and wife in a nuclear family. We can fit our model to other contexts by re-interpreting *w_i_* and *h_i_*. For example, in an Indian context where younger married women live in households with their husband and his parents, inlaws may well be instigators or perpetrators of violence themselves (Bentley, 2018). In such a context, we can re-interpret *w_i_* to refer to young, married women and *h_i_* to refer to their husband and in-laws jointly.

3 Note, we are purely choosing parameter values in example sections to facilitate presentation of our argument: they are not derived from real-world empirical data.

4 Our model of empathetic altruism here matches economic conceptions of ‘pure altruism’ (Andreoni, 1990). ‘Impure altruism’ or ‘warm glow giving’ where the altruistic behaviour is motivated by intrinsic psychological benefits of warmth and fellow-feeling is better modelled as a form of process-based altruism (Andreoni, 1990).

6 For presentation purposes, we assume women also ‘match’ their own efforts at rate *ρ* using the term $\frac{1}{2}\rho \sum_{k}e_{ki}^{2}$. The equilibria do not change if we omit this, but the relevant formulae become more difficult to interpret.

7 Our model of process-based altruism fixes a direct relationship between intrinsic benefit and effort, so there is no variation in this respect in Fig. 6.

8 Nonetheless, rural US communities have also been described as lacking in anonymity and confidentiality and even perpetuating violence against women through fraternal ol’ boy networks that shield perpetrators from punishment (Anderson et al., 2014). ‘Face culture’ has also been argued to protect against sexual aggression in contexts where men expect such behaviour to become publicly known and thus cause them shame (Hall et al., 2005). It may be difficult to make general statements about cultures where social norms concerning domestic violence do or do not exist.

9 Complex ethical concerns surround programmes with a focus on men’s collective action to prevent violence against women, including risks of shifting funding away from programmes that support survivors of violence, delegitimising women-only or women-focused programmes, or marginalising women’s voices by placing men in leadership positions (Macomber, 2018). Masculinity researchers argue these concerns can be addressed in men’s engagement programmes (Flood, 2015), but emphasise the need to dispel ideas that men necessarily benefit from dismantling patriarchy, that men are best placed to talk to other men about domestic violence, and that men fitting the masculine ideal wield most influence over other men’s attitudes to gender equality.
