# Supplementary material for: Modelling collective action to change social norms around domestic violence: social dilemmas and the role of altruism
Source: Humanit Soc Sci Commun. Author manuscript; Available in PMC 2021 Sep 21. (PMC7611687; doi:10.1057/s41599-021-00730-z)
Supplement: Appendix [file EMS133570-supplement-Appendix.docx]

Modelling collective action to change social norms around domestic violence in communities: social dilemmas and the role of altruism

**Technical Appendix**

Lu Gram^1*^, Rolando Granados^1^, Eva Krockow^2^, Nayreen Daruwalla^3^, David Osrin^1^

* Corresponding author, e-mail: [lu.gram.13@ucl.c.uk](mailto:lu.gram.13@ucl.c.uk)

1 – Institute for Global Health

University College London

London WC1N 1EH

United Kingdom

2 - Department of Neuroscience, Psychology and Behaviour

University of Leicester

Leicester LE1 7RH

United Kingdom

3 - Prevention of Violence against Women and Children

Society for Nutrition, Education and Health Action (SNEHA)

Mumbai 400 054

India

## Proof of Proposition 1

*Necessary conditions for a subgame perfect equilibrium.* We find the necessary conditions for a subgame perfect equilibrium through backwards induction. First, consider male strategy selection. Take the derivative of men’s utility function in equation (1) with respect to $v_{i}$ and set $\frac{\partial U_{h_{i}}}{\partial v_{i}}=0$. Rearranging, this yields the following unique solution:

|  | $v_{i}^{*}=\frac{1}{c_{i}}\left( n_{ii}a_{i}\left( e_{i1},\ldots,e_{in} \right)+\sum_{j\neq i} n_{ij}a_{j}\left( e_{j1},\ldots,e_{jn} \right) \right)$ | (A1) |
| --- | --- | --- |

Thus, conditional on women’s efforts $e_{ij}$, a unique dominant strategy exists for men $v_{i}^{*}.$

Knowing this, women choose strategies to maximize their utility based on $v_{i}^{*}(e_{11},\ldots,e_{nn})$.

Differentiating equation (2) in the main text with respect to $e_{ji}$ and re-arranging yields:

| $e_{ji}^{*}=-\frac{s_{i}\left( 1-t_{i} \right)}{d_{ji}}\frac{\partial v_{i}^{*}}{\partial e_{ji}}=-\frac{s_{i}\left( 1-t_{i} \right)n_{ij}}{d_{ji}c_{i}}\frac{\partial a_{j}}{\partial e_{ji}}$ | (A2) |
| --- | --- |

The second equality follows from taking the derivative of (A1) with respect to $e_{ji}$ and substituting the result back into (A2). Under Assumption IV, $\frac{\partial^{2}a_{j}}{\partial^{2}e_{ji}}\geq0$so the right-hand side decreases or stays constant in $e_{ji}$. Since the left-hand side strictly increases in $e_{ji}$, the result is a maximum point. Under Assumption IV, $\frac{\partial a_{j}}{\partial e_{ji}}<0$, so the right-hand side is always strictly positive. Thus, no equilibrium exists where $e_{ji}^{*}=0$. Thus, $v_{i}^{*}$ and $e_{ji}^{*}$ must satisfy (A1) and (A2) in any subgame perfect Nash equilibrium.

*Uniqueness of the subgame perfect equilibrium.* (A2) describes a system of equations for $i,j=1\ldots n$. Since $\frac{\partial a_{j}}{\partial e_{ji}}$ only depends on $e_{j1},\ldots,e_{jn}$, we can separate the full set of equations into discrete bundles of equations indexed by $j$:

| $e_{j1}^{*}=-\frac{s_{1}\left( 1-t_{1} \right)n_{1j}}{d_{j1}c_{1}}\frac{\partial a_{j}}{\partial e_{j1}}\left( e_{j1}^{*},\ldots,e_{jn}^{*} \right)$ |  |
| --- | --- |
| $e_{j2}^{*}=-\frac{s_{2}\left( 1-t_{2} \right)n_{2j}}{d_{j2}c_{2}}\frac{\partial a_{j}}{\partial e_{j2}}\left( e_{j1}^{*},\ldots,e_{jn}^{*} \right)$ |  |
| … |  |
| $e_{jn}^{*}=-\frac{s_{n}\left( 1-t_{n} \right)n_{nj}}{d_{jn}c_{n}}\frac{\partial a_{j}}{\partial e_{jn}}\left( e_{j1}^{*},\ldots,e_{jn}^{*} \right)$ |  |

Each bundle is a self-contained system of equations that can be solved independently. Their solution corresponds to the stationary points of a function

$$g\left( e_{j1},\ldots,e_{jn} \right)=a_{j}\left( e_{j1},\ldots,e_{jn} \right)+\frac{1}{2}\sum\frac{d_{ji}c_{i}}{s_{i}\left( 1-t_{i} \right)n_{ij}}e_{ji}^{2}$$

which is a smooth, strictly convex function as it is the sum of a smooth, non-strictly convex function ($a_{j}$ by Assumption IV) and a smooth, strictly convex function. But smooth, strictly convex functions have unique stationary points. Thus each bundle of equations has a unique solution; thus the whole system of equations (A2) has a unique solution. Substituting the values $e_{ji}^{*}$ into (A1) yields the values for $v_{i}^{*}$.

*Existence of the subgame perfect equilibrium.* Assume there exists a constant $M>0$ such that $\left| \frac{\partial a_{j}}{\partial e_{ji}} \right|<M$ for all $i,j$. Let $K=Mmax\{\frac{s_{i}(1-t_{i})n_{ij}}{d_{i}c_{i}}:i,j\in1..n\}$. Let $\mathcal{B=}\left[ 0,K \right]^{n^{2}}$ and $f:B\to B$ such that

$$f_{ji}\left( e_{11},\ldots,e_{nn} \right)=-\frac{s_{i}(1-t_{i})n_{ij}}{d_{ji}c_{i}}\frac{\partial a_{j}}{\partial e_{ji}}(e_{j1},\ldots,e_{jn})$$

where $f_{ji}$ refers to the $j\left( n-1 \right)+i$^th^ element of $f\left( e_{11},\ldots,e_{nn} \right)$. $f$ is a continuous function from a convex compact set $\mathcal{B}$ to itself. By Brouwer’s fixed point theorem, there is a point $e\mathcal{\in B}$ solving $e=f\left( e \right).$ The individual components of $e$ solve the set of simultaneous equations in (A2). Inserting the resulting value into (A1) yields equilibrium values for $v_{i}$. Thus, a subgame perfect Nash equilibrium exists.

## Proof of Corollary 1

We use implicit differentiation. Recall, $\frac{\partial a_{j}}{\partial e_{ji}}\left( e_{j1}\ldots e_{jn} \right)=\alpha h_{ji}\left( e_{j1}\ldots e_{jn} \right),$ $s_{i}=\sigma\tilde{s}_{i}$, $t_{i}=\tau\tilde{t}_{i}$, $d_{i}={\gamma\tilde{d}}_{i}$, $c_{i}=\kappa\tilde{c_{i}}$, and $n_{ij}=\upsilon\tilde{n}_{ij}$. Define constant $m=\frac{\sigma\alpha\upsilon}{\gamma\kappa}>0$ and rewrite (A2) as

$$e_{ji}^{*}=-\frac{s_{i}\left( 1-t_{i} \right)n_{\mathrm{ij}}}{d_{\mathrm{ji}}c_{i}}\frac{\partial a_{j}}{\partial e_{\mathrm{ji}}}=-m\frac{\tilde{s}_{i}(1-\tau\tilde{t}_{i})}{\tilde{d}_{ji}}\frac{\tilde{n}_{ij}}{\tilde{c}_{i}}h_{ji}\left( e_{j1}^{*},...,e_{jn}^{*} \right)$$

Treat $e_{ji}^{*}$ as a function of $m$. Take the derivative with respect to $m$ on both sides of the equation and rearrange to get:

$$m\tilde{s}_{i}(1-\tau\tilde{t}_{i})\tilde{n}_{ij}\sum_{k} \frac{\partial h_{ji}}{\partial e_{jk}}\frac{\partial e_{jk}^{*}}{\partial m}+\tilde{d}_{ji}\tilde{c}_{i}\frac{\partial e_{ji}^{*}}{\partial m}=-\tilde{s}_{i}(1-\tau\tilde{t}_{i})\tilde{n}_{ij}h_{ji}$$

for all $i,j\in1\ldots n$.

We can write this in matrix form as

| $mSNH\vec{e}+DC\vec{e}=-SN\vec{h}$ | (A3) |
| --- | --- |

where $H=\left[ \begin{matrix} H_{1} & 0 & \cdots& 0 \\ 0 & H_{2} & \cdots& 0 \\ \vdots& \vdots& \ddots& \vdots\\ 0 & 0 & \cdots& H_{n} \end{matrix} \right], H_{i}=\left[ \begin{matrix} \frac{\partial h_{i1}}{\partial e_{i1}} & \cdots& \frac{\partial h_{i1}}{\partial e_{in}} \\ \vdots& \ddots& \vdots\\ \frac{\partial h_{in}}{\partial e_{i1}} & \cdots& \frac{\partial h_{in}}{\partial e_{in}} \end{matrix} \right]$, $\vec{e}=\left[ \begin{matrix} \frac{\partial e_{11}^{*}}{\partial m} \\ \frac{\partial e_{12}^{*}}{\partial m} \\ \vdots\\ \frac{\partial e_{nn}^{*}}{\partial m} \end{matrix} \right]$ and$\vec{h}=\left[ \begin{matrix} h_{11} \\ h_{12} \\ \vdots\\ h_{1n} \\ h_{21} \\ \vdots\\ h_{nn} \end{matrix} \right]$

and $S,N,C$ and $D$ are appropriate diagonal scaling matrices:

$S=\left[ \begin{matrix} S_{1} & \cdots& 0 \\ \vdots& \ddots& \vdots\\ 0 & \cdots& S_{n} \end{matrix} \right],S_{i}=\left[ \begin{matrix} \tilde{s}_{i}(1-\tau\tilde{t}_{i}) & \cdots& 0 \\ \vdots& \ddots& \vdots\\ 0 & \cdots& \tilde{s}_{i}(1-\tau\tilde{t}_{i}) \end{matrix} \right]$ ,$N=\left[ \begin{matrix} N_{1} & \cdots& 0 \\ \vdots& \ddots& \vdots\\ 0 & \cdots& N_{n} \end{matrix} \right],N_{i}=\left[ \begin{matrix} \tilde{n}_{i1} & \cdots& 0 \\ \vdots& \ddots& \vdots\\ 0 & \cdots& \tilde{n}_{in} \end{matrix} \right]$, $D=\left[ \begin{matrix} \tilde{d}_{11} & \cdots& 0 \\ \vdots& \ddots& \vdots\\ 0 & \cdots& \tilde{d}_{nn} \end{matrix} \right]$ and $C=\left[ \begin{matrix} C_{1} & \cdots& 0 \\ \vdots& \ddots& \vdots\\ 0 & \cdots& C_{n} \end{matrix} \right],C_{i}=\left[ \begin{matrix} \tilde{c}_{i} & \cdots& 0 \\ \vdots& \ddots& \vdots\\ 0 & \cdots& \tilde{c}_{i} \end{matrix} \right]$

Solving for $\vec{h}$in (A3) yields:

| $\vec{e}=-\left( mSNH+DC \right)^{-1}SN\vec{h}$ | (A4) |
| --- | --- |

Under Assumption IV, $a_{i}\left( e_{i1},\ldots,e_{in} \right)$ are strictly decreasing, convex functions of $e_{ij}$. As the $H_{i}$ are the Hessian matrices of each $a_{i}$, they are positive semi-definite and so is $H$. Under Assumptions I-V, $S,N,C$ and $D$ all have strictly positive diagonal coefficients. Thus, $mSNH+DC$ must be positive definite, and thus invertible, and $\Theta=\left( mSNH+DC \right)^{-1}SN$ must be positive definite too. Now take the derivative of (A1) with respect to $m$. We get:

$$\frac{\partial v_{i}^{*}}{\partial m}=\frac{1}{c_{i}}\sum_{j} \sum_{k} n_{ij}\frac{\partial a_{j}}{\partial e_{jk}}\frac{\partial e_{jk}^{*}}{\partial m}=\frac{\alpha\upsilon}{c_{i}}\sum_{j} \sum_{k} \tilde{n}_{ij}\tilde{h}_{jk} \frac{\partial e_{jk}^{*}}{\partial m}=\frac{\alpha\upsilon}{c_{i}}N\vec{h}^{T}\vec{e}=-\frac{\alpha\upsilon}{c_{i}}N\vec{h}^{T}\Theta\vec{h}<0$$

The last equality uses (A4), the inequality followed from $\Theta$ being positive definite. Now, $v_{i}^{*}$ only indirectly depends on $\sigma$ and $\gamma$ through their effect on $e_{jk}^{*}$, so the effects of $\sigma$ and $\gamma$ on $v_{i}^{*}$ are unambiguous:

$$\frac{\partial v_{i}^{*}}{\partial\sigma}=\frac{\partial v_{i}^{*}}{\partial m}\frac{\partial m}{\partial\sigma}=\frac{\partial v_{i}^{*}}{\partial m}\frac{\alpha\upsilon}{\kappa\gamma}<0 \mathrm{and}\frac{\partial v_{i}^{*}}{\partial\gamma}=\frac{\partial v_{i}^{*}}{\partial m}\frac{\partial m}{\partial\gamma}=-\frac{\partial v_{i}^{*}}{\partial m}\frac{\alpha\upsilon\sigma}{\kappa\gamma^{2}}>0$$

However, $v_{i}^{*}$ directly depends on $\upsilon$ and $\kappa$ as well as indirectly through $e_{jk}^{*}$. Suppose we increase the costliness of violence perpetration for men. Then, we have:

$$\frac{\partial v_{i}^{*}}{\partial\kappa}=\frac{\partial v_{i}^{*}}{\partial m}\frac{\partial m}{\partial\kappa}+\frac{\partial v_{i}^{*}}{\partial\kappa}=-\frac{\partial v_{i}^{*}}{\partial m}\frac{\alpha\upsilon\sigma}{\kappa^{2}\gamma}-\frac{1}{{\kappa^{2}\tilde{c}}_{i}}\sum_{j} n_{ij}a_{j}\left( e_{j1}^{*},\ldots,e_{jn}^{*} \right)=-\frac{\partial v_{i}^{*}}{\partial m}\frac{\alpha\upsilon\sigma}{\kappa^{2}\gamma}-\frac{v_{i}^{*}}{\kappa}$$

The term $-\frac{v_{i}^{*}}{\kappa}$ represents the direct effect of increasing cost of violence perpetration which is negative for $v_{i}^{*}\geq0$. However, the indirect effect, $-\frac{\partial v_{i}^{*}}{\partial m}\frac{\alpha\upsilon\sigma}{\kappa^{2}\gamma}$, is positive, because women anticipate the greater cost of violence perpetration to men and reduce their own efforts at preventing violence correspondingly. The net effect is ambiguous. For a concrete example, see Scenario 2 at the bottom of this document.

The net effect of $\upsilon$ is similarly ambiguous – stronger pro-violent norms increase perpetration of violence when $v_{i}^{*}\geq0$, but also motivate women towards greater effort to prevent it.

Suppose for any fixed set of $e_{ji}$, $\frac{\partial a_{j}}{\partial\alpha}(e_{j1}\ldots e_{jn})<0$. The $a_{j}$ directly depend on $\alpha$ through their partial derivatives, as well as indirectly through $e_{ji}^{*}$ which depend on $m$ in turn. Thus:

$$\frac{\partial v_{i}^{*}}{\partial\alpha}=\frac{\partial v_{i}^{*}}{\partial m}\frac{\partial m}{\partial\alpha}+\frac{\partial v_{i}^{*}}{\partial\alpha}=\frac{\partial v_{i}^{*}}{\partial m}\frac{\upsilon\sigma}{\kappa^{2}\gamma}+\frac{1}{c_{i}}\sum_{j} n_{ij}\frac{\partial a_{j}}{\partial\alpha}<0$$

Finally, we can follow steps almost identical to our proof that $\frac{\partial v_{i}^{*}}{\partial m}<0$ to prove that $\frac{\partial v_{i}^{*}}{\partial\tau}>0$.

## Proof of Theorem 1

Suppose two women $w_{1}$ and $w_{2}$ have equilibrium efforts $e_{i1}^{*}$ and $e_{j2}^{*}$ respectively given by the Nash equilibrium in Proposition 1. We first prove that there exist changes $\delta e_{i1}>0$ and $\delta e_{j2}>0$ to women’s individually rational effort levels $e_{i1}^{*}$ and $e_{j2}^{*}$ that result in strictly positive improvements to their utility functions. We then prove that the same increments to $e_{i1}^{*}$ and $e_{j2}^{*}$ result in strictly positive improvements to women’s welfare functions.

*Pareto optimal improvements to utility.* Let their utility functions be $U_{w_{1}}$ and $U_{w_{2}}$ respectively. Since we are only considering changes to $e_{i1}$ and $e_{j2}$ we can consider the women’s utilities to be functions of only those two variables, $U_{w_{1}}(e_{i1}, e_{j2})$ and $U_{w_{2}}(e_{i1}, e_{j2}).$ Due to Assumption IV, the utility functions are infinitely smooth. Thus, we can apply Taylor’s theorem to obtain:

$$U_{w_{1}}\left( e_{i1}^{*}+\delta e_{i1},e_{j2}^{*}+\delta e_{j2} \right)=U_{w_{1}}\left( e_{i1}^{*},e_{j2}^{*} \right)+\frac{\partial U_{w_{1}}}{\partial e_{i1}}\left( e_{i1}^{*},e_{j2}^{*} \right)\delta e_{i1}+\frac{\partial U_{w_{1}}}{\partial e_{j2}}\left( e_{i1}^{*},e_{j2}^{*} \right)\delta e_{j2}+R_{1}$$

$$U_{w_{2}}\left( e_{i1}^{*}+\delta e_{i1},e_{j2}^{*}+\delta e_{j2} \right)=U_{w_{2}}\left( e_{i1}^{*},e_{j2}^{*} \right)+\frac{\partial U_{w_{2}}}{\partial e_{i1}}\left( e_{i1}^{*},e_{j2}^{*} \right)\delta e_{i1}+\frac{\partial U_{w_{2}}}{\partial e_{j2}}\left( e_{i1}^{*},e_{j2}^{*} \right)\delta e_{j2}+R_{2}$$

where $R_{1}$ and $R_{2}$ are remainder terms whose size are bounded by $M(\delta e_{i1}^{2}+\delta e_{j2}^{2})$ for a positive constant $M$ when $\delta e_{i1}$ and $\delta e_{j2}$ are sufficiently close to zero (Folland, 2020). Since $e_{i1}^{*}$ and $e_{j2}^{*}$ are utility-maximizing solutions to $U_{w_{1}}$ and $U_{w_{2}}$ respectively, $\frac{\partial U_{w_{1}}}{\partial e_{i1}}\left( e_{i1}^{*},e_{j2}^{*} \right)$ and $\frac{\partial U_{w_{2}}}{\partial e_{j2}}\left( e_{i1}^{*},e_{j2}^{*} \right)$ are zero. Hence, we can re-arrange the above to obtain:

$${\Delta U_{w_{1}}=U}_{w_{1}}\left( e_{i1}^{*}+\delta e_{i1},e_{j2}^{*}+\delta e_{j2} \right)-U_{w_{1}}\left( e_{i1}^{*},e_{j2}^{*} \right)=\frac{\partial U_{w_{1}}}{\partial e_{j2}}\left( e_{i1}^{*},e_{j2}^{*} \right)\delta e_{j2}+R_{1}\geq\frac{\partial U_{w_{1}}}{\partial e_{j2}}\left( e_{i1}^{*},e_{j2}^{*} \right)\delta e_{j2}-M\left( \delta e_{i1}^{2}+\delta e_{j2}^{2} \right)$$

$${\Delta U_{w_{2}}=U}_{w_{2}}\left( e_{i1}^{*}+\delta e_{i1},e_{j2}^{*}+\delta e_{j2} \right)-U_{w_{2}}\left( e_{i1}^{*},e_{j2}^{*} \right)=\frac{\partial U_{w_{2}}}{\partial e_{i1}}\left( e_{i1}^{*},e_{j2}^{*} \right)\delta e_{i1}+R_{2}\geq\frac{\partial U_{w_{2}}}{\partial e_{i1}}\left( e_{i1}^{*},e_{j2}^{*} \right)\delta e_{i1}-M\left( \delta e_{i1}^{2}+\delta e_{j2}^{2} \right)$$

Differentiating equation (2) in the main text with respect to $e_{j2}$, we get $\frac{\partial U_{w_{1}}}{\partial e_{j2}}\left( e_{i1}^{*},e_{j2}^{*} \right)$ = $-s_{1}(1-t_{1})\frac{\partial v_{1}^{*}}{\partial e_{j2}}$. Using equation (A2), this equals $-\frac{s_{1}(1-t_{1})n_{1j}}{c_{1}}\frac{\partial a_{j}}{\partial e_{j2}}$. Under Assumptions I-V, this is strictly positive. Similarly, we can show that $\frac{\partial U_{2}}{\partial e_{i1}}\left( e_{i1}^{*},e_{j2}^{*} \right)>0.$

Assume without loss of generality that $\frac{\partial U_{w_{1}}}{\partial e_{j2}}\left( e_{i1}^{*},e_{j2}^{*} \right)\leq\frac{\partial U_{w_{2}}}{\partial e_{i1}}\left( e_{i1}^{*},e_{j2}^{*} \right)$. Choose $\delta e_{i1}$ and $\delta e_{j2}$ so that $\frac{\varepsilon}{3M}\frac{\partial U_{w_{1}}}{\partial e_{j2}}\left( e_{i1}^{*},e_{j2}^{*} \right)<\delta e_{j2}<\delta e_{i1}<\frac{\sqrt{\varepsilon}}{3M}\frac{\partial U_{w_{1}}}{\partial e_{j2}}\left( e_{i1}^{*},e_{j2}^{*} \right)$ for some small $0<\varepsilon<1$. Now:

$$\frac{\partial U_{w_{2}}}{\partial e_{i1}}\left( e_{i1}^{*},e_{j2}^{*} \right)\delta e_{i1}-M\left( \delta e_{i1}^{2}+\delta e_{j2}^{2} \right)\geq\frac{\partial U_{w_{1}}}{\partial e_{j2}}\left( e_{i1}^{*},e_{j2}^{*} \right)\delta e_{j2}-M\left( \delta e_{i1}^{2}+\delta e_{j2}^{2} \right)\geq\frac{\varepsilon}{3M}\frac{\partial U_{w_{1}}}{\partial e_{j2}}\left( e_{i1}^{*},e_{j2}^{*} \right)^{2}-\frac{2\varepsilon}{9M}\frac{\partial U_{w_{1}}}{\partial e_{j2}}\left( e_{i1}^{*},e_{j2}^{*} \right)^{2}=\frac{\varepsilon}{9M}\frac{\partial U_{w_{1}}}{\partial e_{j2}}\left( e_{i1}^{*},e_{j2}^{*} \right)^{2}>0$$

We have thus obtained changes $\delta e_{i1}$ and $\delta e_{j2}$ to women’s individually rational effort levels $e_{i1}^{*}$ and $e_{j2}^{*}$ that result in $\Delta U_{1}$,$\Delta U_{2}>0$.

*Pareto optimal improvements to welfare.* Consider the following:

$$U_{w_{i}}=-s_{i}\left( 1-t_{i} \right)v_{i}-\frac{1}{2}\sum_{k} d_{ki}e_{ki}^{2}=-s_{1}v_{i}-\frac{1}{2}\sum_{k} d_{ki}e_{ki}^{2}+s_{i}t_{i}v_{i}=W_{i}+s_{i}t_{i}v_{i}$$

Thus, the change to woman $w_{1}$’s welfare resulting from the above additional effort is

$$\Delta W_{1}=W_{1}\left( e_{i1}^{*}+\delta e_{i1},e_{j2}^{*}+\delta e_{j2} \right)-W_{1}\left( e_{i1}^{*},e_{j2}^{*} \right)=\Delta U_{w_{1}}-{s_{1}t}_{1}\left( v_{1}\left( e_{i1}^{*}+\delta e_{i1},e_{j2}^{*}+\delta e_{j2} \right)-v_{1}\left( e_{i1}^{*},e_{j2}^{*} \right) \right)$$

By Assumption IV, the $a_{j}(e_{j1},\ldots,e_{jn})$ are strictly decreasing in each of their arguments, thus $v_{1}\left( e_{i1}^{*}+\delta e_{i1},e_{j2}^{*}+\delta e_{j2} \right)-v_{1}\left( e_{i1}^{*},e_{j2}^{*} \right)<0$ by (A1). Hence, $\Delta W_{1}>0$. Similarly, $\Delta W_{2}>0$.

## Proof of Proposition 2

Using similar steps to those from Proposition 1, one can easily show that in equilibrium:

|  | $e_{ki}^{*}=b-\frac{s_{i}(1-t_{i})n_{ik}}{d_{ki}c_{i}}\frac{\partial a_{k}}{\partial e_{ki}}$ | (A5) |
| --- | --- | --- |

Taking the derivative of women’s welfare with respect to the intrinsic benefit $b$, we get:

$$\frac{\partial W_{i}}{\partial b}=\frac{\partial}{\partial b}\left( -s_{i}v_{i}^{*}-\frac{1}{2}\sum_{k} d_{ki}{e^{*}}_{ki}^{2} \right)=-\frac{s_{i}}{c_{i}}\sum_{k} \sum_{j} n_{ik}\frac{\partial a_{k}}{\partial e_{kj}}\frac{\partial e_{kj}^{*}}{\partial b}-\sum_{k} d_{ki}e_{ki}^{*}\frac{\partial e_{ki}^{*}}{\partial b}=-\frac{s_{i}}{c_{i}}\sum_{k} \sum_{j} {\tau_{ji}n}_{ik}\frac{\partial a_{k}}{\partial e_{kj}}\frac{\partial e_{kj}^{*}}{\partial b}-b\sum_{k} d_{ki}\frac{\partial e_{ki}^{*}}{\partial b}$$

where $\tau_{ji}=\left\{ \begin{aligned} t_{i} \mathrm{if}j=i \\ 1 \mathrm{if}j\neq i \end{aligned} \right.$. The third equality follows from substituting (A5) into $e_{ki}^{*}$. Setting the result $>0$ and re-arranging yields inequality (6) in the main text.

## Proof of Proposition 3

As in Proposition 2, one can easily show that the equilibrium efforts must now satisfy:

|  | $e_{ki}^{*}=-\left( \frac{s_{i}(1-t_{i})n_{ik}}{d_{ki}c_{i}}+\eta\sum_{j\neq i} \frac{s_{j}n_{jk}}{d_{ki}c_{j}} \right)\frac{\partial a_{k}}{\partial e_{ki}}$ | (A6) |
| --- | --- | --- |

Taking the derivative of women’s welfare again, we get:

$$\frac{\partial W_{i}}{\partial\eta}=-\frac{s_{i}}{c_{i}}\sum_{k} \sum_{j} n_{ik}\frac{\partial a_{k}}{\partial e_{kj}}\frac{\partial e_{kj}^{*}}{\partial\eta}-\sum_{k} d_{ki}e_{ki}^{*}\frac{\partial e_{ki}^{*}}{\partial\eta}=-\frac{s_{i}}{c_{i}}\sum_{k} \sum_{j} {\tau_{ji}n}_{ik}\frac{\partial a_{k}}{\partial e_{kj}}\frac{\partial e_{kj}^{*}}{\partial\eta}+\eta\sum_{k} \sum_{j\neq i} \frac{s_{j}}{c_{j}}n_{jk}\frac{\partial a_{k}}{\partial e_{ki}}\frac{\partial e_{ki}^{*}}{\partial\eta}$$

where $\tau_{ji}=\left\{ \begin{aligned} t_{i} \mathrm{if}j=i \\ 1 \mathrm{if}j\neq i \end{aligned} \right.$. The third equality follows from substituting (A6) into $e_{ki}^{*}$. Setting the result $>0$ and re-arranging terms yields inequality (8) in the main text.

## Proof of Proposition 4

As in Proposition 3, one can show that the equilibrium efforts must now satisfy:

|  | $e_{ki}^{*}=-\frac{s_{i}(1-t_{i})n_{ik}}{d_{ki}c_{i}}\frac{\partial a_{k}}{\partial e_{ki}}+\rho\sum_{j} e_{kj}^{*}$ | (A7) |
| --- | --- | --- |

We can express the above in matrix form as follows:

| $\vec{e}_{k}={-\vec{a}}_{k}+\rho P\vec{e}_{k}$ | (A8) |
| --- | --- |

where $\vec{e}_{k}=\left[ \begin{matrix} e_{k1}^{*} \\ e_{k2}^{*} \\ \vdots\\ e_{kn}^{*} \end{matrix} \right], \vec{a}_{k}=\left[ \begin{matrix} \frac{s_{1}(1-t_{1})n_{1k}}{d_{k1}c_{1}}\frac{\partial a_{k}}{\partial e_{k1}} \\ \vdots\\ \frac{s_{n}\left( 1-t_{n} \right)n_{nk}}{d_{kn}c_{n}}\frac{\partial a_{k}}{\partial e_{kn}} \end{matrix} \right], P=\left[ \begin{matrix} 1 & 1 & 1 & \cdots& 1 \\ 1 & 1 & 1 & \cdots& 1 \\ 1 & 1 & 1 & \cdots& 1 \\ \vdots& \vdots& \vdots& \ddots& \vdots\\ 1 & 1 & 1 & \cdots& 1 \end{matrix} \right]={11}^{T}$ for $1=\left[ \begin{matrix} 1 \\ 1 \\ \vdots\\ 1 \end{matrix} \right]$.

We can solve for $\vec{e}_{k}$ and get the following expression:

| $\vec{e}_{k}={-\left( I-\rho{11}^{T} \right)^{-1}\vec{a}}_{k}=-\left( I+\frac{\rho}{1-n\rho}{11}^{T} \right)\vec{a}_{k}$ | (A9) |
| --- | --- |

Here we used the Sherman-Morrison formula $\left( A+uv^{T} \right)^{-1}=A^{-1}-\frac{A^{-1}uv^{T}A^{-1}}{1+v^{T}A^{-1}u}$ in the second equality. For individual elements of $\vec{e}_{k}$ this means:

| $e_{ki}^{*}=-\frac{s_{i}(1-t_{i})n_{ik}}{d_{ki}c_{i}}\frac{\partial a_{k}}{\partial e_{ki}}-\frac{\rho}{1-n\rho}\sum_{j} \frac{s_{j}{(1-t_{j})n}_{jk}}{d_{kj}c_{j}}\frac{\partial a_{k}}{\partial e_{kj}}$ | (A10) |
| --- | --- |

It can be easily shown that solutions to this system of equations (A10) are only guaranteed to exist when $\rho<\frac{1}{n}$. As such, consider the case $\rho<\frac{1}{n}$. Take the derivative of women’s welfare with respect to $\rho$ to get:

$$\frac{\partial W_{i}}{\partial\rho}=-\frac{s_{i}}{c_{i}}\sum_{k} \sum_{j} n_{ik}\frac{\partial a_{k}}{\partial e_{kj}}\frac{\partial e_{kj}^{*}}{\partial\rho}-\sum_{k} d_{ki}e_{ki}^{*}\frac{\partial e_{ki}^{*}}{\partial\rho}=-\frac{s_{i}}{c_{i}}\sum_{k} \sum_{j} {\tau_{ji}n}_{ik}\frac{\partial a_{k}}{\partial e_{kj}}\frac{\partial e_{kj}^{*}}{\partial\rho}+\frac{\rho}{1-n\rho}\sum_{k} \sum_{j} \frac{s_{j}(1-t_{j})n_{jk}}{c_{j}}\frac{\partial a_{k}}{\partial e_{kj}}\frac{\partial e_{ki}^{*}}{\partial\rho}$$

where $\tau_{ji}=\left\{ \begin{aligned} t_{i} \mathrm{if}j=i \\ 1 \mathrm{if}j\neq i \end{aligned} \right.$. The third equality follows from substituting (A10) into $e_{ki}^{*}$. Setting the result $>0$ and re-arranging terms yields inequality (10) in the main text.

## Calculations for concrete examples

### Scenario 1

The scenario used in the main text sets $a_{j}\left( e_{j1},\ldots,e_{jn} \right)=2-\sum_{i} f_{ji}e_{ji}$ with initial values $f_{ji}=0.1$, $t_{i}=0.5$ and $s_{i}=c_{i}=n_{ji}=d_{ji}=1$. The number of couples is $n=10$. Let $f_{ji}=f$, $s_{i}=s$, $t_{i}=t,c_{i}=c,n_{ji}=\tilde{n}$ and $d_{ji}=d$. Then using (A1) and (A2), we have

| $e_{ji}^{*}=\frac{(1-t)s\tilde{n}f}{dc}$ | (A10) |
| --- | --- |

and

| $v_{i}^{*}=\frac{2\tilde{n}n}{c}-\frac{{(1-t){s\tilde{n}^{2}n}^{2}f}^{2}}{dc^{2}}$ | (A11) |
| --- | --- |

Using the above values for $e_{ji}^{*}$ and $v_{i}^{*}$, we can calculate $W_{i}=-s_{i}v_{i}^{*}-\frac{1}{2}\sum_{j} d_{ji}{e^{2}}_{ji}^{*}$. We get values for Figure 2 by substituting values for $f,s,t, c,\tilde{n}$ and $d$ into the above equations. For example, we calculated the effect of varying the effectiveness of preventive action, $f$, on levels of effort, holding all other variables constant using

$$e_{ji}^{*}=\frac{0.5\times1\times1\times f}{1\times1}=0.5f, v_{i}^{*}=\frac{2\times1\times10}{1}-\frac{{0.5\times1\times1^{2}\times{10}^{2}\times f}^{2}}{1\times1^{2}}=20-50f^{2}$$

### Scenario 2

As in Scenario 1, we can use (A10) and (A11) to calculate the result of changing a single variable, holding all the other variables constant. In Scenario 2, we set $\alpha_{j0}=2,$ $f_{ji}=0.1$, $t_{i}=0.5$ and $d_{ji}=1$ as before, but $s=1.5,\tilde{n}=5$ and $n=20$ instead. Now, looking at the effect of changing the cost of violence for perpetrators, we find:

$$v_{i}^{*}=\frac{2\times5\times20}{c}-\frac{{\left( 1-0.5 \right)\times1.5\times5^{2}\times{20}^{2}\times0.1}^{2}}{1\times c^{2}}=\frac{200}{c}-\frac{75}{c^{2}}$$

Taking the derivative of the above with respect to $c$, we get:

$$\frac{\partial v_{i}^{*}}{\partial c}=-\frac{200}{c^{2}}+\frac{150}{c^{3}}$$

which is positive for $c<0.75$, indicating an increase in equilibrium levels of violence resulting from making perpetration of violence more costly.

### Scenario 3

In Scenario 3, we use the same initial values as in Scenario 1. We then calculate the impact of changing parameters corresponding to intrinsic benefit, empathetic altruism, and reciprocity using equations (A5), (A6), and (A10). For example, the impact of reciprocity is:

$$e_{ki}^{*}=-\frac{1\times\left( 1-0.5 \right)\times1}{1\times1}\times(-1)-\frac{\rho}{1-10\times\rho}\sum_{j} \frac{1\times(1-0.5)\times1}{1\times1}\times(-1)=0.5+\frac{5\rho}{1-10\rho}$$

Given the values of the $e_{ki}^{*}$, we can then use (A1) to find violence and welfare levels.

### Scenario 4

We begin with the parameter values from Scenario 1, in which $a_{j}\left( e_{j1},\ldots,e_{jn} \right)=2-\sum_{i} f_{ji}e_{ji}$, and $f_{ji}=0.1$, $t_{i}=0.5$, $s_{i}=c_{i}=n_{ji}=d_{ji}=1$, and the number of couples is $n=10$. We then perturb each of the $f_{ji}$ for $i,j\in\{1\ldots n\}$ by multiplying them by a factor of $e^{0.3Z_{ji}}$ where $Z_{ji}\sim N(0,1)$ are independently distributed standard normal random variables. 95% of perturbations are likely to lie between $e^{-0.3\times2\times1.96}=0.31$ and $e^{0.3\times2\times1.96}=3.2$. Afterwards, we perturb the $t_{i}$ by drawing random, independent $Z_{i}\sim N(0,1)$ and multiplying the $t_{i}$ by $e^{0.3Z_{i}}.$ Similarly, we perturb the parameters $s_{i},c_{i},n_{ji}$ and $d_{ji}$. After thus ensuring that each of the 10 women and men have unique parameter values, we vary levels of altruism and use equation (A5) for each woman independently to calculate the impact of process-based altruism on effort, violence and welfare levels. Similarly, we use equation (A6) for calculating the impact of empathetic altruism.

## References

Folland, G. B. (2020). *Higher-Order Derivatives and Taylor’s Formula in Several Variables*. <https://sites.math.washington.edu/~folland/Math425/taylor2.pdf>
